# Supplementary material for: Streamlining data-intensive biology with workflow systems
Source: Gigascience. 2021 Jan 13;10(1):giaa140. doi: 10.1093/gigascience/giaa140 (PMC8631065; doi:10.1093/gigascience/giaa140)
Supplement: giaa140_GIGA-D-20-00245_Original_Submission [file giaa140_giga-d-20-00245_original_submission.pdf]

GigaScience

Streamlining Data-Intensive Biology with Workflow Systems

--Manuscript Draft--

**Manuscript Number:**	GIGA-D-20-00245
**Full Title:**	Streamlining Data-Intensive Biology with Workflow Systems
**Article Type:**	Review
**Funding Information:**	Gordon and Betty Moore Foundation (GBMF4551) — Dr. C. Titus Brown<br>STATE AND FEDERAL CONTRACTORS WATER AGENCY (A19- 1844) — Not applicable<br>National Science Foundation (1711984) — Dr. N. Tessa Pierce
**Abstract:**	As the scale of biological data generation has increased, the bottleneck of research has shifted from data generation to analysis. Researchers commonly need to build computational workflows that include multiple analytic tools and require incremental development as experimental insights demand tool and parameter modifications. These workflows can produce hundreds to thousands of intermediate files and results that must be integrated for biological insight. Data-centric workflow systems that internally manage computational resources, software, and conditional execution of analysis steps are reshaping the landscape of biological data analysis, and empowering researchers to conduct reproducible analyses at scale. Adoption of these tools can facilitate and expedite robust data analysis, but knowledge of these techniques is still lacking. Here, we provide a series of practices and strategies for leveraging workflow systems with structured project, data, and resource management to streamline large-scale biological analysis.
**Corresponding Author:**	Nuri Teresa Pierce, Ph.D<br>University of California Davis<br>Davis, CA UNITED STATES
**Corresponding Author Secondary Information:**	
**Corresponding Author's Institution:**	University of California Davis
**Corresponding Author's Secondary Institution:**	
**First Author:**	Taylor Reiter
**First Author Secondary Information:**	
**Order of Authors:**	Taylor Reiter<br>Phillip T. Brooks<br>Luiz Irber<br>Shannon E.K. Joslin<br>Charles M. Reid<br>Camille Scott<br>C. Titus Brown<br>N. Tessa Pierce
**Order of Authors Secondary Information:**	
**Additional Information:**	
**Question**	**Response**
Are you submitting this manuscript to a	No

Powered by Editorial Manager® and ProduXion Manager® from Aries Systems Corporation

special series or article collection?	
**Experimental design and statistics**<br><br>Full details of the experimental design and statistical methods used should be given in the Methods section, as detailed in our Minimum Standards Reporting Checklist. Information essential to interpreting the data presented should be made available in the figure legends.<br><br>Have you included all the information requested in your manuscript?	Yes
**Resources**<br><br>A description of all resources used, including antibodies, cell lines, animals and software tools, with enough information to allow them to be uniquely identified, should be included in the Methods section. Authors are strongly encouraged to cite Research Resource Identifiers (RRIDs) for antibodies, model organisms and tools, where possible.<br><br>Have you included the information requested as detailed in our Minimum Standards Reporting Checklist?	Yes
**Availability of data and materials**<br><br>All datasets and code on which the conclusions of the paper rely must be either included in your submission or deposited in publicly available repositories (where available and ethically appropriate), referencing such data using a unique identifier in the references and in the “Availability of Data and Materials” section of your manuscript.<br><br>Have you have met the above requirement as detailed in our Minimum Standards Reporting Checklist?	Yes

Powered by Editorial Manager® and ProduXion Manager® from Aries Systems Corporation

Powered by Editorial Manager® and ProduXion Manager® from Aries Systems Corporation

Editable Manuscript

Click here to access/download;Manuscript;2020-workflows-08-16-2020.docx

Streamlining Data-Intensive Biology With Workflow Systems

This manuscript (permalink) was automatically generated from dib-lab/2020-workflows-paper@d401e38 on August 5, 2020.

Authors

- **Taylor Reiter** 0000-0002-7388-421X · taylorreiter · ReiterTaylor Department of Population Health and Reproduction, University of California, Davis · Funded by Moore Foundation GBMF4551
- **Phillip T. Brooks** 0000-0003-3987-244X · brooksph · brooksph Department of Population Health and Reproduction, University of California, Davis
- **Luiz Irber** 0000-0003-4371-9659 · luizirber · luizirber Department of Population Health and Reproduction, University of California, Davis · Funded by Moore Foundation GBMF4551
- **Shannon E.K. Joslin** 0000-0001-5470-1193 · shannonekj · IntrprtngGnmcs Department of Animal Science, University of California, Davis · Funded by State and Federal Water Contractors A19-1844
- **Charles M. Reid** 0000-0002-7337-8894 · charlesreid1 Department of Population Health and Reproduction, University of California, Davis · Funded by Moore Foundation GBMF4551
- **Camille Scott** 0000-0001-8822-8779 · camillescott · camille_codon Department of Population Health and Reproduction, University of California, Davis · Funded by Moore Foundation GBMF4551
- **C. Titus Brown** 0000-0001-6001-2677 · ctb · ctitusbrown Department of Population Health and Reproduction, University of California, Davis · Funded by Moore Foundation GBMF4551
- **N. Tessa Pierce** 0000-0002-2942-5331 · bluegenes · saltyscientist Department of Population Health and Reproduction, University of California, Davis · Funded by NSF 1711984

Abstract

As the scale of biological data generation has increased, the bottleneck of research has shifted from data generation to analysis. Researchers commonly need to build computational workflows that include multiple analytic tools and require incremental development as experimental insights demand tool and parameter modifications. These workflows can produce hundreds to thousands of intermediate files and results that must be integrated for biological insight. Data-centric workflow systems that internally manage computational resources, software, and conditional execution of analysis steps are reshaping the landscape of biological data analysis, and empowering researchers to conduct reproducible analyses at scale. Adoption of these tools can facilitate and expedite robust data analysis, but knowledge of these techniques is still lacking. Here, we provide a series of practices and strategies for leveraging workflow systems with structured project, data, and resource management to streamline large-scale biological analysis.

Author Summary

We present a guide for workflow-enabled biological sequence data analysis, developed through our own teaching, training and analysis projects. We recognize that this is based on our own use cases and experiences, but we hope that our guide will contribute to a larger discussion within the open source and open science communities and lead to more comprehensive resources. Our main goal is to accelerate the research of scientists conducting sequence analyses by introducing them to organized workflow practices that not only benefit their own research but also facilitate open and reproducible science.

Introduction

Biological research has become increasingly computational. In particular, genomics has experienced a deluge of high-throughput sequencing data that has already reshaped our understanding of the diversity and function of organisms and communities, building basic understanding from ecosystems to human health. The analysis workflows used to produce these insights often integrate hundreds of steps and involve a myriad of decisions ranging from small-scale tool and parameter choices to larger-scale design decisions around data processing and statistical analyses. Each step relies not just on analysis code written by the researcher, but on third-party software, its dependencies, and the compute infrastructure and operating system on which the code is executed. Historically, this has led to the patchwork availability of underlying code for analyses as well as a lack of interoperability of the resulting software and analysis pipelines across compute systems [1]. Combined with unmet training needs in biological data analysis, these conditions undermine the reuse of data and the reproducibility of biological research, vastly limiting the value of our generated data [2].

The biological research community is strongly committed to addressing these issues, recently formalizing the FAIR practices: the idea that all life sciences research (including data and analysis workflows) should be Findable, Accessible, Interoperable, and Reusable [3]. For computational analyses, these ideals are readily achievable with current technology, but implementing them in practice has proven difficult, particularly for biologists with little training in computing [3]. However, the recent maturation of data-centric workflow systems designed to automate and facilitate computational workflows is expanding our capacity to conduct end-to-end FAIR analyses [5]. These workflow systems are designed to handle some aspects of computational workflows internally: namely, the interactions with software and computing infrastructure, and the ordered execution of each step of an analysis. By reducing the manual input and monitoring required at each analysis juncture, these integrated systems ensure that analyses are repeatable and can be executed at much larger scales. In concert, the standardized information and syntax required

for rule-based workflow specification makes code inherently modular and more easily transferable between projects [5,6]. For these reasons, workflow systems are rapidly becoming the workhorses of modern bioinformatics.

Adopting workflow systems requires some level of up-front investment, first to understand the structure of the system, and then to learn the workflow-specific syntax. These challenges can preclude adoption, particularly for researchers without significant computational experience [4]. In our experiences with both research and training, these initial learning costs are similar to those required for learning more traditional analysis strategies, but then provide a myriad of additional benefits that both facilitate and accelerate research. Furthermore, online communities for sharing reusable workflow code have proliferated, meaning the initial cost of encoding a workflow in a system is mitigated via use and re-use of common steps, leading to faster time-to-insight [5,7].

Building upon the rich literature of “best” and “good enough” practices for computational biology [8,9,10], we present a series of strategies and practices for adopting workflow systems to streamline data-intensive biology research. This manuscript is designed to help guide biologists towards project, data, and resource management strategies that facilitate and expedite reproducible data analysis in their research. We present these strategies in the context of our own experiences working with high-throughput sequencing data, but many are broadly applicable to biologists working beyond this field.

Workflows facilitate data-intensive biology

Data-intensive biology typically requires that researchers execute computational workflows using multiple analytic tools and apply them to many experimental samples in a systematic manner. These workflows commonly produce hundreds to thousands of intermediate files and require incremental changes as experimental insights demand tool and parameter modifications. Many intermediate steps are central to the biological analysis, but others, such as converting between file formats, are rote

computational tasks required to passage data from one tool to the next. Some of these steps can fail silently, producing incomplete intermediate files that imperceptively invalidate downstream results and biological inferences. Properly managing and executing all of these steps is vital, but can be both time-consuming and error-prone, even when automated with scripting languages such as bash.

The emergence and maturation of workflow systems designed with bioinformatic challenges in mind has revolutionized computing in data intensive biology [11]. Workflow systems contain powerful infrastructure for workflow management that can coordinate runtime behavior, self-monitor progress and resource usage, and compile reports documenting the results of a workflow (**Figure 1**). These features ensure that the steps for data analysis are minimally documented and repeatable from start to finish. When paired with proper software management, fully-contained workflows are scalable, robust to software updates, and executable across platforms, meaning they will likely still execute the same set of commands with little investment by the user after weeks, months, or years.

Figure 1: ***Workflow Systems:*** *Bioinformatic workflow systems have built-in functionality that facilitates and simplifies running analysis pipelines.* ***A. Samples:*** *Workflow systems enable you to use the same code to run each step on each sample. Samples can be easily added if the analysis expands.* ***B. Software Management:*** *Integration with software management tools (e.g. conda, singularity, docker) can automate software installation for each step.* ***C. Branching, D. Parallelization, and E. Ordering:*** *Workflow systems handle conditional execution, ensuring that tasks are executed in the correct order for each sample file, including executing independent steps in parallel if possible given the resources provided.* ***F. Standard Steps:*** *Many steps are now considered "standard" (e.g. quality control). Workflow languages keep all information for a step together and can be written to enable you to remix and reuse individual steps across pipelines.* ***G. Rerun as necessary:*** *Workflow systems keep track of which steps executed properly and on which samples, and allow you to rerun failed steps (or additional steps) rather than re-*

executing the entire workflow. ***H. Reporting:*** *Workflow languages enable comprehensive reporting on workflow execution and resource utilization by each tool.* ***I. Portability:*** *Analyses written in workflow languages (with integrated software management) can be run across computing systems without changes to code.*

To properly direct an analysis, workflow systems need to encode information about the relationships between every workflow step. In practice, this means that each analysis step must specify the input (or types of inputs) needed for that step, and the output (or types of outputs) being produced. This structure provides several additional benefits. First, workflows become minimally self-documented, as the directed graph produced by workflow systems can be exported and visualized, producing a graphical representation of the relationships between all steps in a pipeline (see **Figure 5**). Next, workflows are more likely to be fully enclosed without undocumented steps that are executed by hand, meaning analyses are more likely to be reproducible. Finally, each step becomes a self-contained unit that can be used and re-used across multiple analysis workflows, so scientists can spend less time implementing standard steps, and more time on their specific research questions. In sum, the internal scaffolding provided by workflow systems helps build analyses that are generally better documented, repeatable, transferable, and scalable.

Getting started with workflows

The workflow system you choose will be largely dependent on your analysis needs. Here, we draw a distinction between two types of workflows: “research” workflows that are under iterative development to answer novel scientific questions, and “production” workflows, which have reached maturity and are primarily used to run a standard analysis on new samples. In particular, research workflows require flexibility and assessment at every step: outliers and edge cases may reveal interesting biological differences, rather than sample processing or technical errors. Many workflow systems can be used for either type, but we note cases where their properties facilitate one of these types over the other.

Using workflows without learning management systems While the benefits of encoding a workflow in a workflow system are immense, the learning curve associated with implementing complete workflows in a new syntax can be daunting. It is possible to obtain the benefits of workflow systems without learning a workflow system. Websites like Galaxy, Cavatica, and EMBL-EBI MGnify offer online portals in which users build workflows around publicly-available or user-uploaded data [12,13,14]. On the command line, many research groups have used workflow systems to build user-friendly pipelines that do not require learning or working with the underlying workflow software. These tools are specified in an underlying workflow language, but are packaged in a user-friendly command-line script that coordinates and executes the workflow. Rather than writing each workflow step, the user can specify data and parameters in a configuration file to customize the run. Some examples include the nf-core RNA-seq pipeline [1,15], the ATLAS metagenome assembly and binning pipeline [16,17], the Sunbeam metagenome analysis pipeline [18,19], and two from our own lab, the dammit eukaryotic transcriptome annotation pipeline [20] and the elvers *de novo* transcriptome pipeline [21]. These tools allow users to take advantage of the benefits of workflow software without needing to invest in curating and writing their own pipeline. The majority of these workflows are production-level workflows designed to execute a series of standard steps, but many provide varying degrees of customizability ranging from tool choice to parameter specification.

Choosing a workflow system If your use case extends beyond these tools, there are several scriptable workflow systems that offer comparable benefits for carrying out your own data-intensive analyses. Each has it own strengths, meaning each workflow software will meet an individuals computing goals differently (see **Table 1**). Our lab has adopted Snakemake, in part due to its similarity and integration with Python, its flexibility for building and testing new analyses in different languages, and its intuitive integration with software management tools (described below)[22]. Snakemake and Nextflow are commonly used for devloping new research pipelines, where flexibility and iterative, branching development is a key feature [23]. Common Workflow Language (CWL) and Workflow Description

Language (WDL) are workflow specification formats that are more geared towards scalability, making them ideal for production-level pipelines with hundreds of thousands of samples [24]. WDL and CWL are commonly executed on platforms such as Terra [25] or Seven Bridges Platform [26]. Language-specific workflow systems, such as ROpenSci's Drake [27], are limited in the scope of tasks they can execute, but are powerful within their language and easier to integrate for those comfortable with that language.

Table 1: Four of the most widely used bioinformatics workflow systems (2020), with links to documentation, example workflows, and general tutorials. In many cases, there may be tutorials online that are tailored for use cases in your field. All of these systems can interact with tools or tasks written in other languages and can function across cloud computing systems and high-performance computing clusters. Some can also import full workflows from other specification languages.

**Workflow System**	**Documentation**	**Example Workflow**	**Tutorial**
Snakemake	snakemake.readthedocs.io	https://github.com/snakemake-workflows/chipseq	https://snakemake.readthedocs.io/en/stable/tutorial/tutorial.html
Nextflow	www.nextflow.io	https://github.com/nf-core/sarek	https://www.nextflow.io/docs/latest/getstarted.html
Common workflow language	www.commonwl.org	https://github.com/EBI-Metagenomics/pipeline-v5	https://www.commonwl.org/user_guide/02-1st-example/index.html
Workflow description language	openwdl.org	https://github.com/gatk-workflows/gatk4-data-processing	https://support.terra.bio/hc/en-us/articles/360037127992–1-howto-Write-your-first-WDL-script-running-GATK-HaplotypeCaller

The best workflow system to choose may be the one with a strong and accessible local or online community in your field, somewhat independent of your computational needs. The availability of field-specific data analysis code for reuse and modification can facilitate the adoption process, as can community support for new users. Fortunately, the standardized syntax required by workflow systems, combined with widespread adoption in the open science community, has resulted in a proliferation of open access workflow-system code for routine analysis steps [28,29]. At the same time, consensus approaches for data analysis are emerging, further encouraging reuse of existing code [30,31,32,33,34].

The Getting started developing workflows section contains strategies for modifying and developing workflows for your own analyses.

Wrangling Scientific Software

Analysis workflows commonly rely on multiple software packages to generate final results. These tools are heterogeneous in nature: they are written by researchers working in different coding languages, with varied approaches to software design and optimization, and often for specific analysis goals. Each program has a number of other programs it depends upon to function ("dependencies"), and as software changes over time to meet research needs, the results may change, even when run with identical parameters. As a result, it is critical to take an organized approach to installing, managing, and keeping track of software and software versions. To meet this need, most workflow managers integrate with software management systems like conda, singularity, and docker [11,35,36].

Software management systems perform some combination of software installation, management, and packaging that alleviate problems that arise from dependencies and facilitate documentation of software versions. On many compute systems, system-wide software management is overseen by system administrators, who ensure commonly-used and requested software is installed into a "module" system available to all users. Unfortunately, this system does not lend itself well for exploring new workflows and software, as researchers do not have permission to install software themselves. The Conda package manager has emerged as a leading solution, largely because it handles both cluster permission and version conflict issues with a user-based software environment system, and features a straightforward "recipe" system which simplifies the process of making new software installable (**Figure 2**). Conda enables lightweight software installation and can be used with the same commands across platforms, but can still be impacted by differences in the host operating system. Alternatively, wrapping software environments in "containers" that capture and reproduce all other aspects of the runtime environment can enhance

reproducibility over time [3]. Container-based software installation via docker and singularity is common for production-level workflows.

Figure 2: ***The conda package and environment manager simplifies software installation and management. A. Conda Recipe Repositories:*** *Each program distributed via Conda has a "recipe" describing all software dependencies needed for installation using Conda (each of which must also be installable via Conda). Recipes are stored and managed in the cloud in separate "channels", some of which specialize in particular fields or languages (e.g. the "bioconda" channel specializes in bioinformatic software, the "r" channel specializes in R language packages) [11].* ***B. Use Conda Environments to Avoid Installation Conflicts:*** *Conda does not require root privileges for software installation, thus enabling use by researchers working on shared cluster systems. However, even user-based software installation can encounter dependency conflicts. For example, you might need to use python2 to install and run a program (e.g. older scripts written by members of your lab), while also using snakemake to execute your workflows (requires python>=3.5). By installing each program into an isolated "environment" that contains only the software required to run that program, you can ensure all programs used throughout your analysis will run without issue. Using small, separate environments for your software and building many simple environments to accommodate different steps in your workflow also reduces the amount of time it takes conda to resolve dependency conflicts between different software tools ("solve" an environment). Conda virtual environments can be created and installed either on the command line, or via an environment YAML file, as shown. In this case, the environment file also specifies which Conda channels to search and download programs from. When specified in a YAML file, conda environments are easily transferable between computers and operating systems. Further, because the version of each package installed in an environment is recorded, workflow reproducibility is enhanced. Although portions of Conda may be superseded by alternative solutions [37], this model of software installation and management will likely persist.*

Getting started with software management

Using software without learning management systems While package managers and containers greatly increase reproducibility, there are a number of ways to test software before needing to worry about installation. Some software packages are available as web-based tools and through a series of data upload and parameter specifications, allow the user to interact with a tool that is running on a back-end server. Integrated development environments (IDE) like PyCharm and RStudio can manage software installation for language-specific tools, and can be very helpful when writing analysis code. These approaches are ideal for testing a tool to determine whether it produces useful output on your data before integration with your reproducible workflow.

Integrating software management within workflows Workflow systems provide seamless integration with software management tools. Each workflow system requires different specification for initiation of software management, but typically requires about one additional line of code per step that requires the use of software. If the software management tool is installed locally, the workflow will automatically download and install the specified environment or container and use it for specified step.

In our experience, the complete solution for using scientific software involves starting with a combination of interactive and exploratory analyses in IDEs and local conda installation to develop an analysis strategy and create an initial workflow. This is then followed by workflow-integrated software management via conda, singularity, or docker for executing the resulting workflow on many samples.

Workflow-Based Project Management

Project management, the strategies and decisions used to keep a project organized, documented, functional, and shareable, is foundational to any research program. Clear organization and management is a learned skill that takes time to implement. Workflow systems both simplify and improve computational

project management, but even workflows that are fully specified in workflow systems require additional investment to stay organized, documented, and backed up.

Systematically document your workflows

Pervasive documentation provides indispensable context for biological insights derived from an analysis, facilitates transparency in research, and increases reusability of the analysis code. Good documentation covers all aspects of a project, including file and results organization, clear and commented code, and accompanying explanatory documents for design decisions and metadata. Workflow systems facilitate building this documentation, as each analysis step (with chosen parameters) and the links between those steps are completely specified within the workflow syntax. This feature streamlines code documentation, particularly if you include as much of the analysis as possible within the automated workflow framework. Outside of the analysis itself, applying consistent organizational design can capitalize on the structure and automation provided by workflows to simplify the generation of quality documentation for all aspects of your project. Below, we discuss project management strategies for building reproducible workflow-enabled biological analyses.

Use consistent, self-documenting names

Using consistent and descriptive identifiers for your files, scripts, variables, workflows, projects, and even manuscripts helps keep your projects organized and interpretable for yourself and collaborators. For workflow systems, this strategy can be implemented by tagging output files with a descriptive identifier for each analysis step, either in the filename or by placing output files within a descriptive output folder. For example, the file shown in **Figure 3** has been preprocessed with a quality control trimming step. For large workflows, placing results from each step of your analysis in isolated, descriptive folders can be essential for keeping your project workspace clean and organized.

Figure 3: **Consistent and informative file naming improves organization and interpretability**. For ease of grouping and referring to input files, it is useful to keep unique sample identification in the filename, often with a metadata file explaining the meaning of each unique descriptor. For analysis scripts, it can help to implement a numbering scheme, where the name of first file in the analysis begins with “00”, the next with “01”, etc. For output files, it can help to add a short, unique identifier to output files processed with each analysis step. This particular file is a RAD sequencing fastq file of a fish species that has been preprocessed with a fastq quality trimming tool.

Store workflow metadata with the workflow

Developing biological analysis workflows can involve hundreds of small decisions: What parameters work best for each step? Why did you use a certain reference file for annotation as compared with other available files? How did you finally manage to get around the program or installation error? All of these pieces of information contextualize your results and may be helpful when sharing your findings. Keeping information about these decisions in an intuitive and easily accessible place helps you find it when you need it. To capitalize on the utility of version control systems described below, it is most useful to store this information in plain text files. Each main directory of a project should include notes on the data or scripts contained within, so that a collaborator could look into the directory and understand what to find there (especially since that “collaborator” is likely to be you, a few months from now!). Code itself can contain documentation - you can include comments with the reasoning behind algorithm choice or include a link to the seqanswers post that helped you decide how to shape your differential expression analysis. Larger pieces of information can be kept in “README” or notes documents kept alongside your code and other documents. For example, a GitHub repository documenting the reanalysis of the Marine Microbial Eukaryote Transcriptome Sequencing Project uses a README alongside the code to document

the workflow and digital object identifiers for data products [38,39]. While this particular strategy cannot be automated, it is critical for interpreting the final results of your workflow.

Document data and analysis exploration using computational notebooks

Computational notebooks allow users to combine narrative, code, and code output (e.g. visualizations) in a single location, enabling the user to conduct analysis and visually assess the results in a single file (see **Figure 4**). These notebooks allow for fully documented iterative analysis development, and are particularly useful for data exploration and developing visualizations prior to integration into a workflow or as a report generated by a workflow that can be shared with collaborators.

Figure 4: ***Examples of computational notebooks.*** *Computational notebooks allow the user to mix text, code, and results in one document.* ***Panel A.*** *shows an RMarkdown document viewed in the RStudio integrated development environment, while* ***Panel B.*** *shows a rendered HTML file produced by knitting the RMarkdown document [40].* ***Panel C.*** *shows a Jupyter Notebook, where code, text, and results are rendered inline as each code chunk is executed [41]. The second grey chunk is a raw Markdown chunk with text that will be rendered inline when executed. Both notebooks generate a histogram of a metadata feature, number of generations, from a long-term evolution experiment with Escherichia coli [42]. Computational notebooks facilitate sharing by packaging narrative, code, and visualizations together. Computational notebooks can be further packaged with tools like Binder [43]. Binder builds an executable environment (capable of running RStudio and Jupyter notebooks) out of a GitHub repository using package management systems and docker to build reproducible and executable software environments as specified in the repository. Binders can be shared with collaborators (or students in a classroom setting), and analysis and visualization can be ephemerally reproduced or altered from the code provided in computational notebooks.*

Visualize your workflow

Visual representations can help illustrate the connections in a workflow and improve the readability and reproducibility of your project. At the highest level, flowcharts that detail relationships between steps of a workflow can help provide big-picture clarification, especially when the pipeline is complicated. For individual steps, a graphical representation of the output can show the status of the project or provide insight on additional analyses that should be added. For example, **Figure 5** exhibits a modified Snakemake workflow visualization from an RNA-seq quantification pipeline [44].

Figure 5: A directed acyclic graph (DAG) that illustrates connections between all steps of a sequencing data analysis workflow. Each box represents a step in the workflow, while lines connect sequential steps. The DAG shown in this figure illustrates a real bioinformatics workflow for RNA-seq quantification was generated by modifying the default Snakemake workflow DAG. While the workflow is complex, it is coordinated by a workflow system that alleviates the need for a user to directly manage file interdependencies.

Version control your project

As your project develops, version control allows you to keep track of changes over time. You may already do this in some ways, perhaps with frequent hard drive backups or by manually saving different versions of the same file - e.g. by appending the date to a script name or appending "version_1" or "version_FINAL" to a manuscript draft. For computational workflows, using version control systems such as Git or Mercurial can be used to keep track of all changes over time, even across multiple systems, scripting languages, and project contributors (see **Figure 6**). If a key piece of a workflow inexplicably stops working, consistent version control can allow you to rewind in time and identify differences from when the pipeline worked to when it stopped working. Backing up your version controlled analysis in an

online repository such as GitHub, GitLab, or Bitbucket provides critical insurance as you iteratively modify and develop your workflow.

Figure 6: ***Version Control*** *Version control systems (e.g. Git, Mercurial) work by storing incremental differences in files from one saved version ("commit") to the next. To visualize the differences between each version, text editors such as Atom and online services such as GitHub, GitLab and Bitbucket use red highlighting to denote deletions, and green highlighting to denote additions. In this trivial example, a typo in version 1 (in red) was corrected in version 2 (in green). These systems are extremely useful for code and manuscript development, as it is possible to return to the snapshot of any saved version. This means that version control systems save you from accidental deletions, preserve code you thought you no longer needed and preserve a record of project changes over time.*

When combined with online backups, version control systems also facilitate code and data availability and reproducibility for publication. For example, to preserve the version of code that produced published results, you can create a "release": a snapshot of the current code and files in a GitHub repository. You can then generate a digital object identifier (DOI) for that release using a permanent documentation service such as Zenodo ([45]) and make it available to reviewers and beyond (see "sharing" section, below).

Share your workflow and analysis code

Sharing your workflow code with collaborators, peer reviewers, and scientists seeking to use a similar method can foster discussion and review of your analysis. Sticking to a clear documentation strategy, using a version control system, and packaging your code in notebooks or as a workflow prepare them to be easily shared with others. To go one step further, you can package your code with tools like Binder, ReproZip, or Whole Tale, or make interactive visualizations with tools like Shiny apps or Plotly. These

approaches let others run the code on cloud computers in environments identical to those in which the original computation was performed (**Figure 4**, **Figure 7**) [43,46,47]. These tools substantially reduce overhead associated with interacting with code and data, and in doing so, make it fast and easy to rerun portions of the analysis, check accuracy, or even tweak the analysis to produce new results. If you also share your code and workflows publicly, you will also help contribute to the growing resources for open workflow-enabled biological research.

*Figure 7**: Interactive visualizations facilitate sharing and repeatability. A.** Interactive visualization dashboard in the Pavian Shiny app for metagenomic analysis [48,49]. Shiny allows you to build interactive web pages using R code. Data is manipulated by R code in real-time in a web page, producing analysis and visualizations of a data set. Shiny apps can contain user-specifiable parameters, allowing a user to control visualizations or analyses. As seen above, sample "PT1" is selected, and taxonomic ranks class and order are excluded. Shiny apps allow collaborators who may or may not know R to modify R visualizations to fit their interests. **B.** Plotly heatmap of transcriptional profiling in human brain samples [50]. Hovering over a cell in the heatmap displays the sample names from the x and y axis, as well as the intensity value. Plotting tools like plotly and vega-lite produce single interactive plots that can be shared with collaborators or integrated into websites [51,52]. Interactive visualizations are also helpful in exploratory data analysis.*

Getting started developing workflows

In our experience, the best way to have your workflow system work *for* you is to include as much of your analysis as possible within the automated workflow framework, use self-documenting names, include analysis visualizations, and keep rigorous documentation alongside your workflow that enables you to understand each decision and entirely reproduce any manual steps. Some of the tools discussed above will

inevitably change over time, but these principles apply broadly and will help you design clear, well-documented, and reproducible analyses. Ultimately, you will need to experiment with strategies that work for you – what is most important is to develop a clear set of strategies and implement them tenaciously. Below, we provide a few practical strategies to try as you begin developing your own workflows.

Start with working code When building a workflow for the first time, creating an initial workflow based on a subset of your sample data can help verify that the workflow, tools, and command line syntax function at a basic level. This functioning example code then provides a reliable workflow framework free of syntax errors which you can customize for your data without the overhead of generating correct workflow syntax from scratch. **Table 1** provides links to official repositories containing tutorials and example biological analysis workflows, and workflow tutorials and code sharing websites like GitHub, GitLab, and Bitbucket have many publicly available workflows for other analyses. If a workflow is available through Binder, you can test and experiment with workflow modification on Binder's cloud system without needing to install a workflow manager or software management tool on your local compute system [43].

Test with subsampled data While a workflow may run on test data, this is not a guarantee it will run on all data. After verifying your chosen example workflow is functional, try running it with your own data or some public data related to your species or condition of interest. If your analysis allows, trying the workflow on a small subset of the data first can save time, energy, and computational resources. For example, if working with FASTQ data, you can subsample the first million lines of a file (first 250k reads) by running:

```
head -n 1000000 FASTQ_FILE.fq > test_fastq.fq
```

While there are many more sophisticated ways to subsample reads, this technique should be sufficient for testing each step of a most workflows prior to running your full dataset. In specific cases, such as eukaryotic genome assembly, you may need to be more intentional with how you subsample reads.

Document your process Document your changes, explorations, and errors as you develop. We recommend using the Markdown language so your documentation is in plain text to facilitate version control, but can still include helpful visual headings, code formatting, and embedded images. Markdown editors with visual previewing, such as HackMD, can greatly facilitate notetaking, and Markdown documents are visually rendered properly within your online version control backups on services such as GitHub [53].

Develop your workflow From your working code, iteratively modify and add workflow steps to meet your data analysis needs. This strategy allows you to find and fix mistakes on small sections of the workflow. Periodically clean your output directory and rerun the entire workflow, to ensure all steps are fully interoperable (using small test data will improve the efficiency of this step!). If possible, using mock or control datasets can help you verify that the analysis you are building actually returns correct biological results. Tutorials and tool documentation are useful companions during development; as with any language, remembering workflow-specific syntax takes time and practice.

Assess your results Evaluate your workflow results as you go. Consider what aspects (e.g. tool choice, program parameters) can be evaluated rigorously, and assess each step for expected behavior. Other aspects (e.g. filtering metadata, joining results across programs or analysis, software and workflow bugs) will be more difficult to evaluate. Wherever possible, set up positive and negative controls to ensure your analysis is performing the desired analysis properly. If you're certain an analysis is executing as designed, tracking down unusual results may reveal interesting biological differences.

Back up early and often As you write new code, back up your changes in an online repository such as GitHub, GitLab, or Bitbucket. These services support both drag-and-drop and command line interaction. Data backup will be discussed in the next section, Data and resource management for workflow-enabled biology.

Scale up your workflow Bioinformatic tools vary in the resources they require: some analysis steps are compute-intensive, other steps are memory intensive, and still others will have large intermediate storage needs. If using high-performance computing system or the cloud, you will need to request resources for running your pipeline, often provided as a simultaneous execution limit or purchased by your research group on a cost-per-compute basis. Workflow systems provide built-in tools to monitor resource usage for each step. Running a complete workflow on a single sample with resource monitoring enabled generates an estimate of computational resources needed for each step. These estimates can be used to set appropriate resource limits for each step when executing the workflow on your remaining samples. Strategies for resource management will be addressed in the next section, Data and resource management for workflow-enabled biology.

Find a community and ask for help when you need it Local and online users groups are helpful communities when learning a workflow language. When you are first learning, help from more advanced users can save you hours of frustration. After you've progressed, providing that same help to new users can help you cement the syntax in your mind and tackle more advanced uses. Data-centric workflow systems have been enthusiastically adopted by the open science community, and as a consequence, there is a critical mass of tutorials and open access code, as well as code discussion on forums and via social media, particularly Twitter. Post in the relevant workflow forums when you have hit a stopping point you are unable to work through. Be respectful of people's time and energy and be sure to include appropriate details important to your problem (see Strategic troubleshooting section).

Data and resource management for workflow-enabled biology

Advancements in sequencing technologies have greatly increased the volume of data available for biological query [54]. Workflow systems, by virtue of automating many of the time-intensive project

management steps traditionally required for data-intensive biology, can increase our capacity for data analysis. However, conducting biological analyses at this scale requires a coordinated approach to data and computational resource management. Below, we provide recommendations for data acquisition, management, and quality control that have become especially important as the volume of data has increased. Finally, we discuss securing and managing appropriate computational resources for the scale of your project.

Managing large-scale datasets

Experimental design, finding or generating data, and quality control are quintessential parts of data intensive biology. There is no substitute for taking the time to properly design your analysis, identify appropriate data, and conduct sanity checks on your files. While these tasks are not automatable, many tools and databases can aid in these processes.

Look for appropriate publicly-available data

With vast amounts of sequencing data already available in public repositories, it is often possible to begin investigating your research question by seeking out publicly available data. In some cases, these data will be sufficient to conduct your entire analysis. In others cases, particularly for biologists conducting novel experiments, these data can inform decisions about sequencing type, depth, and replication, and can help uncover potential pitfalls before they cost valuable time and resources.

Most journals now require data for all manuscripts to be made accessible, either at publication or after a short moratorium. Further, the FAIR (findable, accessible, interoperable, reusable) data movement has improved the data sharing ecosystem for data-intensive biology [55,56,57,58,59,60,60,61]. You can find relevant sequencing data either by starting from the “data accessibility” sections of papers relevant to your research or by directly searching for your organism, environment, or treatment of choice in public data portals and repositories. The International Nucleotide Sequence Database Collaboration (INSDC),

which includes the Sequence Read Archive (SRA), European Nucleotide Archive (ENA), and DataBank of Japan (DDBJ) is the largest repository for raw sequencing data, but no longer accepts sequencing data from large consortia projects [62]. These data are instead hosted in consortia-specific databases, which may require some domain-specific knowledge for identifying relevant datasets and have unique download and authentication protocols. For example, raw data from the Tara Oceans expedition is hosted by the Tara Ocean Foundation [63]. Additional curated databases focus on processed data instead, such as gene expression in the Gene Expression Omnibus (GEO) [64]. Organism-specific databases such as **Wormbase** (*Caenorhabditis elegans*) specialize on curating and integrating sequencing and other data associated with a model organism [65]. Finally, rather than focusing on certain data types or organisms, some repositories are designed to hold any data and metadata associated with a specific project or manuscript (e.g. Open Science Framework, Dryad, Zenodo [66]).

Consider analysis when generating your own data

If generating your own data, proper experimental design and planning are essential. For cost-intensive sequencing data, there are a range of decisions about experimental design and sequencing (including sequencing type, sequencing depth per sample, and biological replication) that impact your ability to properly address your research question. Conducting discussions with experienced bioinformaticians and statisticians, **prior to beginning your experiments** if possible, is the best way to ensure you will have sufficient statistical power to detect effects. These considerations will be different for different types of sequence analysis. To aid in early project planning, we have curated a series of domain-specific references that may be useful as you go about designing your experiment (see **Table 2**). Given the resources invested in collecting samples for sequencing, it’s important to build in a buffer to preserve your experimental design in the face of unexpected laboratory or technical issues. Once generated, it is always a good idea to have multiple independent backups of raw sequencing data, as it typically cannot be easily regenerated if lost to computer failure or other unforeseeable events.

Table 2: ***References for experimental design and considerations for common sequencing chemistries***.

**Sequencing type**	**Resources**
RNA-sequencing	[30,67,68]
Metagenomic sequencing	[31,69,70]
Amplicon sequencing	[71,72,73]
Microbial isolate sequencing	[74]
Eukaryotic genome sequencing	[75,76,77,78]
Whole-genome resequencing	[79]
RAD-sequencing	[80,80,81,82,83,84]
single cell RNA-seq	[85,86]

As your experiment progresses, keep track of as much information as possible: dates and times of sample collection, storage, and extraction, sample names, aberrations that occurred during collection, kit lot used for extraction, and any other sample and sequencing measurements you might be able to obtain (temperature, location, metabolite concentration, name of collector, well number, plate number, machine your data was sequenced, on etc). This metadata allows you to keep track of your samples, to control for batch effects that may arise from unintended batching during sampling or experimental procedures and makes the data you collect reusable for future applications and analysis by yourself and others. Wherever possible, follow the standard guidelines for formatting metadata for scientific computing to limit downstream processing and simplify analyses requiring these metadata (see: [10]). We have focused here on sequencing data; for data management over long-term ecological studies, we recommend [87].

Getting started with sequencing data

Protect valuable data

Aside from the code itself, raw data are the most important files associated with a workflow, as they cannot be regenerated if accidentally altered or deleted. Keeping a read-only copy of raw data alongside a workflow as well multiple backups protects your data from accidents and computer failure. This also removes the imperative of storing intermediate files as these can be easily regenerated by the workflow.

When sharing or storing files and results, data version control can keep track of differences in files such as changes from tool parameters or versions. The version control tools discussed in the Workflow-based project management section are primarily designed to handle small files, but repositories such as the Open Science Framework, Figshare, Zenodo, and Dryad can be used for storing larger files and datasets.

The Open Science Framework (OSF; [66]) is a free service that provides powerful collaboration and sharing tools, provides built-in version control, integrates with other storage and version control repositories, guarantees data preservation, and enables you to keep projects private until they are ready to share. Like other services geared towards data sharing, OSF also enables generation of a digital object identifier (doi) for each project. While other services such as Git Large File Storage (LFS), Figshare [88], Zenodo [45], and the Dryad Digital Repository [89] each provide important services for sharing and version control, OSF provides the most comprehensive set of free tools for managing data storage and backup. As free tools often limit the size of files that can be stored, a number of cloud backup and storage services are also available for purchase or via university contract, including Google Drive, Box, Dropbox, Amazon Web Services, and Backblaze. Full computer backups can be conducted to these storage locations with tools like rclone [90].

Ensure data integrity during transfers

If you're working with publicly-available data, you may be able to work on a compute system where the data are already available, circumventing time and effort required for downloading and moving the data. Databases such as the Sequence Read Archive (SRA) are now available on commercial cloud computing systems, and open source projects such as Galaxy enable working with SRA sequence files directly from a web browser [12,91]. Ongoing projects such as the NIH Common Fund Data Ecosystem aim to develop a data portal to make NIH Common Fund data, including biomedical sequencing data, more findable, accessible, interoperable, and reusable (FAIR).

In most cases, you'll still need to transfer some data - either downloading raw data or transferring important intermediate and results files for backup and sharing (or both). Transferring compressed files (gzip, bzip2, BAM/CRAM, etc.) can improve transfer speed and save space, and checksums can be used to to ensure file integrity after transfer (see **Figure 8**).

Figure 8: ***Use Checksums to ensure file integrity*** *Checksum programs (e.g. md5, sha256) encode file size and content in a single value known as a "checksum". For any given file, this value will be identical across platforms when calculated using the same checksum program. When transferring files, calculate the value of the checksum prior to transfer, and then again after transfer. If the value is not identical, there was an error introduced during transfer (e.g. file truncation, etc). Checksums are often provided alongside publicly available files, so that you can verify proper download. Tools like rsync and rclone that automate file transfers use checksums internally to verify that files were transferred properly, and some GUI file transfer tools (e.g. Cyberduck) can assess checksums when they are provided [90]. If you generated your own data and receieved sequencing files from a sequencing center, be certain you also receive a checksum for each of your files to ensure they download properly.*

Perform quality control at every step

The quality of your input data has a major impact on the quality of the output results, no matter whether your workflow analyzes six samples or six hundred. Assessing data at every analysis step can reveal problems and errors early, before they waste valuable time and resources. Using quality control tools that provide metrics and visualizations can help you assess your datasets, particularly as the size of your input data scales up. However, data from different species or sequencing types can produce anomalous quality control results. You are ultimately the single most effective quality control tool that you have, so it is important to critically assess each metric to determine those that are relevant for your particular data.

Look at your files Quality control can be as simple as looking at the first few and last few lines of input and output data files, or checking the size of those files (see **Table 3**). To develop an intuition for what proper inputs and outputs look like for a given tool, it is often helpful to first run the test example or data that is packaged with the software. Comparing these input and output file formats to your own data can help identify and address inconsistencies.

Table 3: ***Some bash commands are useful to quickly explore the contents of a file.*** *By using these commands, the user can detect common formatting problems or other abnormalities.*

**command**	**function**	**example**
ls -lh	list files with information in a human-readable format	ls -lh *fastq.gz
head	print the first 6 lines of a file to standard out	head samples.csv
tail	print the last 6 lines of a file to standard out	tail samples.csv
less	show the contents of a file in a scrollable screen	less samples.csv
zless	show the contents of a gzipped file in a scrollable screen	zless sample1.fastq.gz
wc -l	count the number of lines in a file	wc -l ecoli.fasta
cat	print a file to standard out	cat samples.csv

grep	find matching text and print the line to standard out	grep “>” ecoli.fasta
cut	cut columns from a table	cut -d“,” -f1 samples.csv

Visualize your data Visualization is another powerful way to pick out unusual or unexpected patterns. Although large abnormalities may be clear from looking at files, others may be small and difficult to find. Visualizing raw sequencing data with FastQC (**Figure 9A**) and processed sequencing data with tools like the Integrative Genome Viewer and plotting tabular results files using python or R can make aberrant or inconsistent results easier to track down [93,94].

Figure 9: ***Visualizations produced by MultiQC.*** *MultiQC finds and automatically parses log files from other tools and generates a combined report and parsed data tables that include all samples. MultiQC currently supports 88 tools.* ***A.*** *MultiQC summary of FastQC Per Sequence GC Content for 1905 metagenome samples. FastQC provides quality control measurements and visualizations for raw sequencing data from a single sample, and is a near-universal first step in sequencing data analysis because of the insights it provides [93,94]. FastQC measures and summarizes 10 quality metrics and provides recommendations for whether an individual sample is within an acceptable quality range. Not all metrics readily apply to all sequencing data types. For example, while multiple GC peaks might be concerning in whole genome sequencing of a bacterial isolate, we would expect a non-normal distribution for some metagenome samples that contain organisms with diverse GC content. Samples like this can be seen in red in this figure.* ***B.*** *MultiQC summary of Salmon quant reads mapped per sample for RNA-seq samples [95]. In this figure, we see that MultiQC summarizes the number of reads mapped and percent of reads mapped, two values that are reported in the Salmon log files.*

Pay attention to warnings and log files Many tools generate log files or messages while running. These files contain information about the quantity, quality, and results from the run, or error messages about why a run failed. Inspecting these files can be helpful to make sure tools ran properly and consistently, or

to debug failed runs. Parsing and visualizing log files with a tool like MultiQC can improve interpretability of program-specific log files (**Figure 9** [96]).

Look for common biases in sequencing data Biases in sequencing data originate from experimental design, methodology, sequencing chemistry, or workflows, and are helpful to target specifically with quality control measures. The exact biases in a specific data set or workflow will vary greatly between experiments so it is important to understand the sequencing method you have chosen and incorporate appropriate filtration steps into your workflow. For example, PCR duplicates can cause problems in libraries that underwent an amplification step, and often need to be removed prior to downstream analysis [97,98,99,100,101].

Check for contamination Contamination can arise during sample collection, nucleotide extraction, library preparation, or through sequencing spike-ins like PhiX, and could change data interpretation if not removed [102,103,104]. Libraries sequenced with high concentrations of free adapters or with low concentration samples may have increased barcode hopping, leading to contamination between samples [105].

Consider the costs and benefits of stringent quality control for your data Good quality data is essential for good downstream analysis. However, stringent quality control can sometimes do more harm than good. For example, depending on sequencing depth, stringent quality trimming of RNA-sequencing data may reduce isoform discovery [106]. To determine what issues are most likely to plague your specific data set, it can be helpful to find recent publications using a similar experimental design, or to speak with experts at a sequencing core.

Because sequencing data and applications are so diverse, there is no one-size-fits-all solution for quality control. It is important to think critically about the patterns you expect to see given your data and your biological problem, and consult with technical experts whenever possible.

Securing and managing appropriate computational resources

Sequence analysis requires access to computing systems with adequate storage and analysis power for your data. For some smaller-scale datasets, local desktop or even laptop systems can be sufficient, especially if using tools that implement data-reduction strategies such as minhashing [107]. However, larger projects require additional computing power, or may be restricted to certain operating systems (e.g. linux). For these projects, solutions range from research-focused high performance computing systems to research-integrated commercial analysis platforms. Both research-only and and commercial clusters provide avenues for research and educational proposals to enable access to their computing resources (see **Table 4**). In preparing for data analysis, be sure to allocate sufficient computational resources and funding for storage and analysis, including large intermediate files and resources required for personnel training. Note that workflow systems can greatly facilitate faithful execution of your analysis across the range of computational resources available to you, including distribution across cloud computing systems.

Table 4: ***Computing Resources*** *Bioinformatic projects often require additional computing resources. If a local or university-run high-performance computing cluster is not available, computing resources are available via a number of grant-based or commercial providers.*

**Provider**	**Access Model**	**Restrictions**
Amazon Web Services	Paid	
Bionimbus Protected Data Cloud	Research allocation	users with eRA commons account
Cyverse Atmosphere	Free with limits	storage and compute hours
EGI federated cloud	Access by contact	European partner countries
Galaxy	Free with storage limits	data storage limits

Google Cloud Platform	Paid	
Google Colab	Free	computational notebooks, no resource guarantees
Microsoft Azure	Paid	
NSF XSEDE	Research allocation	USA researchers or collaborators
Open Science Data Cloud	Research allocation	
Wasabi	Paid	data storage solution only

Getting started with resource management

As the scale of data increases, the resources required for analysis can balloon. Bioinformatic workflows can be long-running, require high-memory systems, or involve intensive file manipulation. Some of the strategies below may help you manage computational resources for your project.

Apply for research units if eligible There are a number of cloud computing services that offer grants providing computing resources to data-intensive researchers (**Table 4**). In some cases, the resources provided may be sufficient to cover your entire analysis.

Develop on a local computer when possible Since workflows transfer easily across systems, it can be useful to develop individual analysis steps on a local laptop. If the analysis tool will run on your local system, test the step with subsampled data, such as that created in the Getting started developing workflows section. Once working, the new workflow component can be run at scale on a larger computing system. Workflow system tool resource usage reporting can help determine the increased resources needed to execute the workflow on larger systems. For researchers without access to free or granted computing resources, this strategy can save significant cost.

Gain quick insights using sketching algorithms Understanding the basic structure of data, the relationship between samples, and the approximate composition of each sample can very helpful at the beginning of data analysis, and can often drive analysis decisions in different directions than those originally intended. Although most bioinformatics workflows generate these types of insights, there are a few tools that do so rapidly, allowing the user to generate quick hypotheses that can be further tested by more extensive, fine-grained analyses. Sketching algorithms work with compressed approximate representations of sequencing data and thereby reduce runtimes and computational resources. These approximate representations retain enough information about the original sequence to recapitulate the main findings from many exact but computationally intensive workflows. Most sketching algorithms estimate sequence similarity in some way, allowing you to gain insights from these comparisons. For example, sketching algorithms can be used to estimate all-by-all sample similarity which can be visualized as a Principle Component Analysis or a multidimensional scaling plot, or can be used to build a phylogenetic tree with accurate topology. Sketching algorithms also dramatically reduce the runtime for comparisons against databases (e.g. all of GenBank), allowing users to quickly compare their data against large public databases.

Rowe 2019 [108] reviewed programs and genomic use cases for sketching algorithms, and provided a series of tutorial workbooks (e.g. Sample QC notebook: [109]).

Use the right tools for your question RNA-seq analysis approaches like differential expression or transcript clustering rely on transcript or gene counts. Many tools can be used to generate these counts by quantifying the number of reads that overlap with each transcript or gene. For example, tools like STAR and HISAT2 produce alignments that can be post-processed to generate per-transcript read counts [110,111]. However, these tools generate information-rich output, specifying per-base alignments for each read. If you are only interested in read quantification, quasi-mapping tools provide the desired results while reducing the time and resources needed to generate and store read count information [112,113].

Seek help when you need it In some cases, you may find that your accessible computing system is ill-equipped to handle the type or scope of your analysis. Depending on the system, staff members may be able to help direct you to properly scale your workflow to available resources, or guide you in tailoring computational unit allocations or purhcases to match your needs.

Strategies for troubleshooting

Workflows, and research software in general, invariably require troubleshooting and iteration. When first starting with a workflow system, it can be difficult to interpret code and usage errors from unfamiliar tools or languages [2]. Further, the iterative development process of research software means functionality may change, new features may be added, or documentation may be out of date [114]. The challenges of learning and interacting with research software require time and patience [4].

One of the largest barriers to surmounting these challenges is learning how, when, and where to ask for help. Below we outline a strategy for troubleshooting that can help build your own knowledge while respecting both your own time and that of research software developers and the larger bioinformatic community. In the "where to seek help" section, we also recommend locations for asking general questions around data-intensive analysis, including discussion of tool choice, parameter selection, and other analysis strategies. Beyond these tips, workshops and materials from training organizations such as the Carpentries, R-Ladies, RStudio can arm you with the tools you need to start troubleshooting and jump-start software and data literacy in your community [115]. Getting involved with these workshops and communities not only provides educational benefits but also networking and career-building opportunities.

How to help yourself: Try to pinpoint your issue or error

Software errors can be the result of syntax errors, dependency issues, operating system conflicts, bugs in the software, problems with the input data, and many other issues. Running the software on the provided test data can help narrow the scope of error sources: if the test data successfully runs, the command is likely free of syntax errors, the source code is functioning, and the tool is likely interacting appropriately with dependencies and the operating system. If the test data runs but the tool still produces an error when run with your data and parameters, the error message can be helpful in discovering the cause of the error. In many cases, the error you've encountered has been encountered many times before, and searching for the error online can turn up a working solution. If there is a software issue tracker for the software (e.g. on the GitHub, GitLab, or Bitbucket repository), or a Gitter, Slack, or Google Groups page, performing a targeted search with the error message may provide additional context or a solution for the error. If targeted searches do not return a results, Googling the error message with the program name is a good next step. Searching with several variants and iteratively adding information such as the type of input data, the name of the coding language or computational platform, or other relevant information, can improve the likelihood that a there will be a match. There are a vast array of online resources for bioinformatic help ranging from question sites such as Stack Overflow and BioStars, to personal or academic blogs and even tutorials and lessons written by experts in the field [116]. This increases the discoverability of error messages and their solutions.

Sometimes, programs fail without outputting an error message. In cases like these, the software's help (usually accessible on the command line via tool-name --help) and official documentation may provide clues or additional example use cases that may be helpful in resolving an error. Syntax errors are extremely common, and typos as small as a single, misplaced character or amount of whitespace can affect the code. If a command matches the documentation and appears syntactically correct, the software version (often accessible at the command line tool-name --version) may be causing the error.

Best practices for software development follow “semantic versioning” principles, which aim to keep the arguments and functionality the same for all minor releases of the program (e.g. 1.1 to 1.2) and only change functions with major releases (e.g. 1.x to 2.0).

How to seek help: include the right details with your question

When searching for the error message and reading the documentation do not resolve an error, it is usually appropriate to for seek help either from the software developers or from a bioinformatics community. When asking for help, it’s essential to provide the right details so that other users and developers can understand the exact conditions that produced the error. At minimum, include the name and version of the program, the method used to install it, whether or not the test data ran, the exact code that produced the error, the error message, and the full output text from the run (if any is produced). The type and version of the operating system you are using is also helpful to include. Sometimes, this is enough information for others to spot the error. However, if it appears that there may bug in the underlying code, specifying or providing the minimum amount of data required to reproduce the error (e.g. reproducible example [117,118]) enables other to reproduce and potentially solve the error at hand. Putting the effort into gathering this information both increases your own understanding of the problem and makes it easier and faster for others to help solve your issue. Furthermore, it signals respect for the time that these developers and community members dedicate to helping troubleshoot and solve user issues.

Where to seek help: online and local communities of practice

Online communities and forums are a rich source of archived bioinformatics errors with many helpful community members. For errors with specific programs, often the best place to post is the developers’ preferred location for answering questions and solving errors related to their program. For open source programs on GitHub, GitLab, or Bitbucket, this is often the “Issues” tab within the software repository, but it could alternatively be a Google groups list, gitter page, or other specified forum. Usually, the

documentation indicates the best location questions. If question is more general, such as asking about program choice or workflows, forums relevant to your field such as Stack Overflow, BioStars, or SEQanswers are good choices, as posts here are often seen by a large community of researchers. Before posting, search through related topics to double check the question has not already been answered. As more research software development and troubleshooting is happening openly in online repositories, it is becoming more important than ever to follow a code of conduct that promotes open and harassment-free discussion environment [119]. Look for codes of conduct in the online forums you participate in, and make sure you do your part to help ensure a welcoming community for participants of all backgrounds and computational competencies.

While there is lots of help available online, there is no substitute for local communities. Local communities may come in the form of a tech meetup, a users group, a hacky hour, or an informal meetup of researchers using similar tools. While this may seem like just a local version of Stack Overflow, the local, member-only nature can help create a safe and collaborative online space for troubleshooting problems often encountered by your local bioinformatics community. The benefit to beginners is clear: learning the best way to post questions and the important parts of errors, while getting questions answered so they can move forward in their research. Intermediate users may actually find these communities most useful, as they can also accelerate their own troubleshooting skills by helping others solve issues that they have already struggled through. While it can be helpful to have some experts available to help answer questions or to know when to escalate to Stack Overflow or other communities, a collaborative community of practice with members at all experience levels can help all its members move their science forward faster.

If such a community does not yet exist in your area, building this sort of community (discussed in detail in [120]), can be as simple as hosting a seminar series or starting meetup sessions for data analysis co-working. In our experience, it can also be useful to set up a local online forum (e.g. discourse) for group troubleshooting.

Conclusion

Bioinformatics-focused workflow systems have reshaped data-intensive biology, reducing execution hurdles and empowering biologists to conduct reproducible analyses at the massive scale of data now available. Shared, interoperable research code is enabling biologists to spend less time rewriting common analysis steps, and more time on interesting biological questions. We believe these workflow systems will become increasingly important as dataset size and complexity continue to grow. This manuscript provides a directed set of project, data, and resource management strategies for adopting workflow systems to facilitate and expedite reproducible biological research. While the included data management strategies are tailored to our own experiences in high-throughput sequencing analysis, we hope that these principles enable biologists both within and beyond our field to reap the benefits of workflow-enabled data-intensive biology.

Declarations

Acknowledgements

Thank you to all the members and affiliates of the Lab for Data-Intensive Biology at UC Davis for providing valuable feedback on earlier versions of this manuscript and growing these practices alongside us. We also thank the Carpentries Community for fundamentally shaping many of the ideas and practices we cover in this manuscript.

Author Contributions

Author	Contributions
TER	Conceptualization; Methodology; Writing - Original Draft; Writing - Review and Editing; Visualization
PTB**	Methodology; Writing - Review and Editing
LCI**	Methodology; Writing - Review and Editing
SEJ**	Methodology; Visualization; Writing - Review and Editing
CMR**	Methodology; Writing - Review and Editing
CSW**	Methodology; Writing - Review and Editing
CTB	Methodology; Writing - Review and Editing; Supervision; Funding Acquisition
NTP	Conceptualization; Methodology; Writing - Original Draft; Writing - Review and Editing; Visualization; Supervision; Funding Acquisition

**co-equal contributions

Availability of Data and Materials

All materials for this manuscript can be found at https://github.com/dib-lab/2020-workflows-paper.

Competing Interests

The authors declare that no competing interests exist.

References

1. **The nf-core framework for community-curated bioinformatics pipelines**
Philip A. Ewels, Alexander Peltzer, Sven Fillinger, Harshil Patel, Johannes Alneberg, Andreas Wilm, Maxime Ulysse Garcia, Paolo Di Tommaso, Sven Nahnsen
Nature Biotechnology (2020-02-13) https://doi.org/ggk3qh
DOI: 10.1038/s41587-020-0439-x · PMID: 32055031

2. **Unmet needs for analyzing biological big data: A survey of 704 NSF principal investigators**
Lindsay Barone, Jason Williams, David Micklos
PLOS Computational Biology (2017-10-19) https://doi.org/gcgdcc
DOI: 10.1371/journal.pcbi.1005755 · PMID: 29049281 · PMCID: PMC5654259

3. **Practical Computational Reproducibility in the Life Sciences**
Björn Grüning, John Chilton, Johannes Köster, Ryan Dale, Nicola Soranzo, Marius van den Beek, Jeremy Goecks, Rolf Backofen, Anton Nekrutenko, James Taylor
Cell Systems (2018-06) https://doi.org/ggdv3z
DOI: 10.1016/j.cels.2018.03.014 · PMID: 29953862 · PMCID: PMC6263957

4. **A graduate student perspective on overcoming barriers to interacting with open-source software**
Oihane Cereceda, Danielle E. A. Quinn
FACETS (2020-01-01) https://doi.org/ggw7f3
DOI: 10.1139/facets-2019-0020

5. **Scientific workflows: Past, present and future**
Malcolm Atkinson, Sandra Gesing, Johan Montagnat, Ian Taylor
Future Generation Computer Systems (2017-10) https://doi.org/ggs77s
DOI: 10.1016/j.future.2017.05.041

6. **Rule-based workflow management for bioinformatics**
John S. Conery, Julian M. Catchen, Michael Lynch
The VLDB Journal (2005-09-09) https://doi.org/fhzqvp
DOI: 10.1007/s00778-005-0153-9

7. **Robust Cross-Platform Workflows: How Technical and Scientific Communities Collaborate to Develop, Test and Share Best Practices for Data Analysis**
Steffen Möller, Stuart W. Prescott, Lars Wirzenius, Petter Reinholdtsen, Brad Chapman, Pjotr Prins, Stian Soiland-Reyes, Fabian Klötzl, Andrea Bagnacani, Matúš Kalaš, … Michael R. Crusoe
Data Science and Engineering (2017-11-16) https://doi.org/ggwrrk
DOI: 10.1007/s41019-017-0050-4

8. **Best Practices for Scientific Computing**
Greg Wilson, D. A. Aruliah, C. Titus Brown, Neil P. Chue Hong, Matt Davis, Richard T. Guy, Steven H. D. Haddock, Kathryn D. Huff, Ian M. Mitchell, Mark D. Plumbley, … Paul Wilson
PLoS Biology (2014-01-07) https://doi.org/qtt
DOI: 10.1371/journal.pbio.1001745 · PMID: 24415924 · PMCID: PMC3886731

9. **Computing Workflows for Biologists: A Roadmap**
Ashley Shade, Tracy K. Teal
PLOS Biology (2015-11-24) https://doi.org/f72r9m
DOI: 10.1371/journal.pbio.1002303 · PMID: 26600012 · PMCID: PMC4658184

10. **Good enough practices in scientific computing**
Greg Wilson, Jennifer Bryan, Karen Cranston, Justin Kitzes, Lex Nederbragt, Tracy K. Teal
PLOS Computational Biology (2017-06-22) https://doi.org/gbkbwp
DOI: 10.1371/journal.pcbi.1005510 · PMID: 28640806 · PMCID: PMC5480810

11. **Bioconda: sustainable and comprehensive software distribution for the life sciences**
Björn Grüning, Ryan Dale, Andreas Sjödin, Brad A. Chapman, Jillian Rowe, Christopher H. Tomkins-Tinch, Renan Valieris, Johannes Köster, The Bioconda Team
Nature Methods (2018-07-02) https://doi.org/gd2xzp
DOI: 10.1038/s41592-018-0046-7 · PMID: 29967506

12. **The Galaxy platform for accessible, reproducible and collaborative biomedical analyses: 2018 update**
Enis Afgan, Dannon Baker, Bérénice Batut, Marius van den Beek, Dave Bouvier, Martin Čech, John

Chilton, Dave Clements, Nate Coraor, Björn A Grüning, … Daniel Blankenberg
Nucleic Acids Research (2018-07-02) https://doi.org/gdwxpr
DOI: 10.1093/nar/gky379 · PMID: 29790989 · PMCID: PMC6030816

13. **Data Commons to Support Pediatric Cancer Research**
Samuel L. Volchenboum, Suzanne M. Cox, Allison Heath, Adam Resnick, Susan L. Cohn, Robert Grossman
American Society of Clinical Oncology Educational Book (2017) https://doi.org/ggv5zs
DOI: 10.14694/edbk_175029 · PMID: 28561664

14. **MGnify: the microbiome analysis resource in 2020**
Alex L Mitchell, Alexandre Almeida, Martin Beracochea, Miguel Boland, Josephine Burgin, Guy Cochrane, Michael R Crusoe, Varsha Kale, Simon C Potter, Lorna J Richardson, … Robert D Finn
Nucleic Acids Research (2019-11-07) https://doi.org/ggctnm
DOI: 10.1093/nar/gkz1035 · PMID: 31696235 · PMCID: PMC7145632

15. **nf-core/rnaseq**
GitHub
https://github.com/nf-core/rnaseq

16. **metagenome-atlas/atlas**
GitHub
https://github.com/metagenome-atlas/atlas

17. **ATLAS: a Snakemake workflow for assembly, annotation, and genomic binning of metagenome sequence data**
Silas Kieser, Joseph Brown, Evgeny M. Zdobnov, Mirko Trajkovski, Lee Ann McCue
bioRxiv (2019-08-20) https://doi.org/ggv5zm
DOI: 10.1101/737528

18. **sunbeam-labs/sunbeam**
GitHub
https://github.com/sunbeam-labs/sunbeam

19. **Sunbeam: an extensible pipeline for analyzing metagenomic sequencing experiments**
Erik L. Clarke, Louis J. Taylor, Chunyu Zhao, Andrew Connell, Jung-Jin Lee, Bryton Fett, Frederic D. Bushman, Kyle Bittinger
Microbiome (2019-03-22) https://doi.org/gf9sd6
DOI: 10.1186/s40168-019-0658-x · PMID: 30902113 · PMCID: PMC6429786

20. **dib-lab/dammit**
GitHub
https://github.com/dib-lab/dammit

21. **dib-lab/elvers**
GitHub
https://github.com/dib-lab/elvers

22. **Snakemake–a scalable bioinformatics workflow engine**
J. Koster, S. Rahmann
Bioinformatics (2012-08-20) https://doi.org/gd2xzq
DOI: 10.1093/bioinformatics/bts480 · PMID: 22908215

23. **Nextflow enables reproducible computational workflows**
Paolo Di Tommaso, Maria Chatzou, Evan W Floden, Pablo Prieto Barja, Emilio Palumbo, Cedric Notredame
Nature Biotechnology (2017-04-11) https://doi.org/gfj52z
DOI: 10.1038/nbt.3820 · PMID: 28398311

24. **Common Workflow Language, v1.0**
Peter Amstutz, Michael R. Crusoe, Nebojša Tijanić, Brad Chapman, John Chilton, Michael Heuer, Andrey Kartashov, Dan Leehr, Hervé Ménager, Maya Nedeljkovich, … Luka Stojanovic
Figshare (2016) https://doi.org/gf6ppg
DOI: 10.6084/m9.figshare.3115156.v2

25. **Terra.Bio**
Terra.Bio
https://terra.bio

26. **The Seven Bridges Platform**
Seven Bridges
https://www.sevenbridges.com/platform/

27. **The drake R package: a pipeline toolkit for reproducibility and high-performance computing**
William Michael Landau
The Journal of Open Source Software (2018-01-26) https://doi.org/gd876m
DOI: 10.21105/joss.00550

28. **Scalable Workflows and Reproducible Data Analysis for Genomics**
Francesco Strozzi, Roel Janssen, Ricardo Wurmus, Michael R. Crusoe, George Githinji, Paolo Di Tommaso, Dominique Belhachemi, Steffen Möller, Geert Smant, Joep de Ligt, Pjotr Prins
Methods in Molecular Biology (2019) https://doi.org/ggv5zh
DOI: 10.1007/978-1-4939-9074-0_24 · PMID: 31278683

29. **"Sequana": a Set of Snakemake NGS pipelines**
Thomas Cokelaer, Dimitri Desvillechabrol, Rachel Legendre, Mélissa Cardon
The Journal of Open Source Software (2017-08-30) https://doi.org/ggv5zt
DOI: 10.21105/joss.00352

30. **A survey of best practices for RNA-seq data analysis**
Ana Conesa, Pedro Madrigal, Sonia Tarazona, David Gomez-Cabrero, Alejandra Cervera, Andrew McPherson, Michał Wojciech Szcześniak, Daniel J. Gaffney, Laura L. Elo, Xuegong Zhang, Ali Mortazavi
Genome Biology (2016-01-26) https://doi.org/f3vhpj
DOI: 10.1186/s13059-016-0881-8 · PMID: 26813401 · PMCID: PMC4728800

31. **Shotgun metagenomics, from sampling to analysis**
Christopher Quince, Alan W Walker, Jared T Simpson, Nicholas J Loman, Nicola Segata
Nature Biotechnology (2017-09-12) https://doi.org/gbv6nf
DOI: 10.1038/nbt.3935 · PMID: 28898207

32. **Current best practices in single-cell RNA-seq analysis: a tutorial**
Malte D Luecken, Fabian J Theis
Molecular Systems Biology (2019-06-19) https://doi.org/gf4f4d
DOI: 10.15252/msb.20188746 · PMID: 31217225 · PMCID: PMC6582955

33. **Next-generation biology: Sequencing and data analysis approaches for non-model organisms**
Rute R. da Fonseca, Anders Albrechtsen, Gonçalo Espregueira Themudo, Jazmín Ramos-Madrigal, Jonas Andreas Sibbesen, Lasse Maretty, M. Lisandra Zepeda-Mendoza, Paula F. Campos, Rasmus Heller, Ricardo J. Pereira
Marine Genomics (2016-12) https://doi.org/f9h2s2
DOI: 10.1016/j.margen.2016.04.012 · PMID: 27184710

34. **Best practices for analysing microbiomes**
Rob Knight, Alison Vrbanac, Bryn C. Taylor, Alexander Aksenov, Chris Callewaert, Justine Debelius, Antonio Gonzalez, Tomasz Kosciolek, Laura-Isobel McCall, Daniel McDonald, … Pieter C. Dorrestein
Nature Reviews Microbiology (2018-05-23) https://doi.org/gdj32p
DOI: 10.1038/s41579-018-0029-9 · PMID: 29795328

35. **Singularity: Scientific containers for mobility of compute**
Gregory M. Kurtzer, Vanessa Sochat, Michael W. Bauer
PLOS ONE (2017-05-11) https://doi.org/f969fz
DOI: 10.1371/journal.pone.0177459 · PMID: 28494014 · PMCID: PMC5426675

36. **Docker: lightweight Linux containers for consistent development and deployment**
Dirk Merkel
Linux Journal (2014-03-01)

37. **TheSnakePit/mamba**
GitHub
https://github.com/TheSnakePit/mamba

38. **dib-lab/dib-MMETSP**
GitHub
https://github.com/dib-lab/dib-MMETSP

39. **Re-assembly, quality evaluation, and annotation of 678 microbial eukaryotic reference transcriptomes**
Lisa K Johnson, Harriet Alexander, C Titus Brown
GigaScience (2019-04) https://doi.org/ggrd6x
DOI: 10.1093/gigascience/giy158 · PMID: 30544207 · PMCID: PMC6481552

40. **R Markdown** https://rmarkdown.rstudio.com/

41. **IOS Press Ebooks - Jupyter Notebooks – a publishing format for reproducible computational workflows** http://ebooks.iospress.nl/publication/42900

42. **Tempo and mode of genome evolution in a 50,000-generation experiment**
Olivier Tenaillon, Jeffrey E. Barrick, Noah Ribeck, Daniel E. Deatherage, Jeffrey L. Blanchard, Aurko Dasgupta, Gabriel C. Wu, Sébastien Wielgoss, Stéphane Cruveiller, Claudine Médigue, … Richard E. Lenski
Nature (2016-08-01) https://doi.org/f8283v
DOI: 10.1038/nature18959 · PMID: 27479321 · PMCID: PMC4988878

43. **Binder 2.0 - Reproducible, interactive, sharable environments for science at scale**
Project Jupyter, Matthias Bussonnier, Jessica Forde, Jeremy Freeman, Brian Granger, Tim Head, Chris Holdgraf, Kyle Kelley, Gladys Nalvarte, Andrew Osheroff, … Carol Willing
SciPy (2018) https://doi.org/gfwcm6
DOI: 10.25080/majora-4af1f417-011

44. **ngs-docs/2020-ggg-201b-rnaseq**
GitHub
https://github.com/ngs-docs/2020-ggg-201b-rnaseq

45. **Zenodo - Research. Shared.** https://zenodo.org/

46. **Computing environments for reproducibility: Capturing the "Whole Tale"**
Adam Brinckman, Kyle Chard, Niall Gaffney, Mihael Hategan, Matthew B. Jones, Kacper Kowalik, Sivakumar Kulasekaran, Bertram Ludäscher, Bryce D. Mecum, Jarek Nabrzyski, … Kandace Turner
Future Generation Computer Systems (2019-05) https://doi.org/ggv5zj
DOI: 10.1016/j.future.2017.12.029

47. **ReproZip**
Fernando Chirigati, Rémi Rampin, Dennis Shasha, Juliana Freire
Association for Computing Machinery (ACM) (2016) https://doi.org/ggxs3d
DOI: 10.1145/2882903.2899401

48. **Pavian** https://fbreitwieser.shinyapps.io/pavian/

49. **Pavian: interactive analysis of metagenomics data for microbiome studies and pathogen identification**
Florian P Breitwieser, Steven L Salzberg
Bioinformatics (2019-09-25) https://doi.org/ggnb3n
DOI: 10.1093/bioinformatics/btz715 · PMID: 31553437

50. **Visualizing Biological Data** https://plotly.com/python/v3/ipython-notebooks/bioinformatics/

51. **Plotly: The front-end for ML and data science models** /

52. **Vega-Lite: A Grammar of Interactive Graphics**
Arvind Satyanarayan, Dominik Moritz, Kanit Wongsuphasawat, Jeffrey Heer

IEEE Transactions on Visualization and Computer Graphics (2017-01) https://doi.org/f92f32
DOI: 10.1109/tvcg.2016.2599030 · PMID: 27875150

53. **HackMD - Collaborative Markdown Knowledge Base**
HackMD
https://hackmd.io

54. **Documentation** https://www.ncbi.nlm.nih.gov/sra/docs/sragrowth/

55. **The FAIR Guiding Principles for scientific data management and stewardship**
Mark D. Wilkinson, Michel Dumontier, IJsbrand Jan Aalbersberg, Gabrielle Appleton, Myles Axton, Arie Baak, Niklas Blomberg, Jan-Willem Boiten, Luiz Bonino da Silva Santos, Philip E. Bourne, … Barend Mons
Scientific Data (2016-03-15) https://doi.org/bdd4
DOI: 10.1038/sdata.2016.18 · PMID: 26978244 · PMCID: PMC4792175

56. **The international nucleotide sequence database collaboration**
Ilene Karsch-Mizrachi, Toshihisa Takagi, Guy Cochrane, on behalf of the International Nucleotide Sequence Database Collaboration
Nucleic Acids Research (2018-01-04) https://doi.org/ggwp7h
DOI: 10.1093/nar/gkx1097 · PMID: 29190397 · PMCID: PMC5753279

57. **Public Microbial Resource Centers: Key Hubs for Findable, Accessible, Interoperable, and Reusable (FAIR) Microorganisms and Genetic Materials**
P. Becker, M. Bosschaerts, P. Chaerle, H.-M. Daniel, A. Hellemans, A. Olbrechts, L. Rigouts, A. Wilmotte, M. Hendrickx
Applied and Environmental Microbiology (2019-10-16) https://doi.org/ggbpc9
DOI: 10.1128/aem.01444-19 · PMID: 31471301 · PMCID: PMC6803313

58. **Linking the International Wheat Genome Sequencing Consortium bread wheat reference genome sequence to wheat genetic and phenomic data**
Michael Alaux, Jane Rogers, Thomas Letellier, Raphaël Flores, Françoise Alfama, Cyril Pommier, Nacer Mohellibi, Sophie Durand, Erik Kimmel, Célia Michotey, … International Wheat Genome Sequencing Consortium
Genome Biology (2018-08-17) https://doi.org/gf23xv
DOI: 10.1186/s13059-018-1491-4 · PMID: 30115101 · PMCID: PMC6097284

59. **FAIR: A Call to Make Published Data More Findable, Accessible, Interoperable, and Reusable**
Leonore Reiser, Lisa Harper, Michael Freeling, Bin Han, Sheng Luan
Molecular Plant (2018-09) https://doi.org/ggwp69
DOI: 10.1016/j.molp.2018.07.005 · PMID: 30076986

60. **The Integrative Human Microbiome Project**
The Integrative HMP (iHMP) Research Network Consortium
Nature (2019-05-29) https://doi.org/gf3wp9
DOI: 10.1038/s41586-019-1238-8 · PMID: 31142853 · PMCID: PMC6784865

61. **The Pfam protein families database in 2019**
Sara El-Gebali, Jaina Mistry, Alex Bateman, Sean R Eddy, Aurélien Luciani, Simon C Potter, Matloob Qureshi, Lorna J Richardson, Gustavo A Salazar, Alfredo Smart, … Robert D Finn
Nucleic Acids Research (2019-01-08) https://doi.org/gfhx7r
DOI: 10.1093/nar/gky995 · PMID: 30357350 · PMCID: PMC6324024

62. **The International Nucleotide Sequence Database Collaboration**
Guy Cochrane, Ilene Karsch-Mizrachi, Toshihisa Takagi, International Nucleotide Sequence Database Collaboration
Nucleic Acids Research (2016-01-04) https://doi.org/gf28sk
DOI: 10.1093/nar/gkv1323 · PMID: 26657633 · PMCID: PMC4702924

63. **Open science resources for the discovery and analysis of Tara Oceans data**
Stéphane Pesant, Fabrice Not, Marc Picheral, Stefanie Kandels-Lewis, Noan Le Bescot, Gabriel Gorsky, Daniele Iudicone, Eric Karsenti, Sabrina Speich, Romain Troublé, … Sarah Searson
Scientific Data (2015-05-26) https://doi.org/gd32xw
DOI: 10.1038/sdata.2015.23 · PMID: 26029378 · PMCID: PMC4443879

64. **Gene Expression Omnibus: NCBI gene expression and hybridization array data repository**
R. Edgar
Nucleic Acids Research (2002-01-01) https://doi.org/fttpkn
DOI: 10.1093/nar/30.1.207 · PMID: 11752295 · PMCID: PMC99122

65. **WormBase: a modern Model Organism Information Resource**
Todd W Harris, Valerio Arnaboldi, Scott Cain, Juancarlos Chan, Wen J Chen, Jaehyoung Cho, Paul Davis, Sibyl Gao, Christian A Grove, Ranjana Kishore, … Paul W Sternberg
Nucleic Acids Research (2019-10-23) https://doi.org/ggv5zk
DOI: 10.1093/nar/gkz920 · PMID: 31642470 · PMCID: PMC7145598

66. **Open Science Framework (OSF)**
Erin D. Foster, MSLS, Ariel Deardorff, MLIS
Journal of the Medical Library Association (2017-04-04) https://doi.org/gfxvhq
DOI: 10.5195/jmla.2017.88 · PMCID: PMC5370619

67. **Erratum: How many biological replicates are needed in an RNA-seq experiment and which differential expression tool should you use?**
Nicholas J. Schurch, Pietà Schofield, Marek Gierliński, Christian Cole, Alexander Sherstnev, Vijender Singh, Nicola Wrobel, Karim Gharbi, Gordon G. Simpson, Tom Owen-Hughes, … Geoffrey J. Barton
RNA (2016-09-16) https://doi.org/ggv5zr
DOI: 10.1261/rna.058339.116 · PMID: 27638913 · PMCID: PMC5029462

68. **Power analysis and sample size estimation for RNA-Seq differential expression**
Travers Ching, Sijia Huang, Lana X. Garmire
RNA (2014-11) https://doi.org/f6nstp
DOI: 10.1261/rna.046011.114 · PMID: 25246651 · PMCID: PMC4201821

69. **Unlocking the potential of metagenomics through replicated experimental design**
Rob Knight, Janet Jansson, Dawn Field, Noah Fierer, Narayan Desai, Jed A Fuhrman, Phil Hugenholtz, Daniel van der Lelie, Folker Meyer, Rick Stevens, … Jack A Gilbert
Nature Biotechnology (2012-06-07) https://doi.org/f32nds
DOI: 10.1038/nbt.2235 · PMID: 22678395 · PMCID: PMC4902277

70. **Contamination in Low Microbial Biomass Microbiome Studies: Issues and Recommendations**
Raphael Eisenhofer, Jeremiah J. Minich, Clarisse Marotz, Alan Cooper, Rob Knight, Laura S. Weyrich
Trends in Microbiology (2019-02) https://doi.org/gfpfkt
DOI: 10.1016/j.tim.2018.11.003 · PMID: 30497919

71. **Consistent and correctable bias in metagenomic sequencing experiments**
Michael R McLaren, Amy D Willis, Benjamin J Callahan
eLife (2019-09-10) https://doi.org/gf7wbr
DOI: 10.7554/elife.46923 · PMID: 31502536 · PMCID: PMC6739870

72. **From Benchtop to Desktop: Important Considerations when Designing Amplicon Sequencing Workflows**
Dáithí C. Murray, Megan L. Coghlan, Michael Bunce
PLOS ONE (2015-04-22) https://doi.org/f7hjz7
DOI: 10.1371/journal.pone.0124671 · PMID: 25902146 · PMCID: PMC4406758

73. **Assessment of variation in microbial community amplicon sequencing by the Microbiome Quality Control (MBQC) project consortium**
Rashmi Sinha, Galeb Abu-Ali, Emily Vogtmann, Anthony A Fodor, Boyu Ren, Amnon Amir, Emma Schwager, Jonathan Crabtree, Siyuan Ma, Christian C Abnet, … The Microbiome Quality Control Project Consortium
Nature Biotechnology (2017-10-02) https://doi.org/gbzrdx
DOI: 10.1038/nbt.3981 · PMID: 28967885 · PMCID: PMC5839636

74. **Completing bacterial genome assemblies: strategy and performance comparisons**
Yu-Chieh Liao, Shu-Hung Lin, Hsin-Hung Lin
Scientific Reports (2015-03-04) https://doi.org/f686w8
DOI: 10.1038/srep08747 · PMID: 25735824 · PMCID: PMC4348652

75. **Earth BioGenome Project: Sequencing life for the future of life**
Harris A. Lewin, Gene E. Robinson, W. John Kress, William J. Baker, Jonathan Coddington, Keith A. Crandall, Richard Durbin, Scott V. Edwards, Félix Forest, M. Thomas P. Gilbert, … Guojie Zhang
Proceedings of the National Academy of Sciences (2018-04-24) https://doi.org/gdh5vz
DOI: 10.1073/pnas.1720115115 · PMID: 29686065 · PMCID: PMC5924910

76. **Opportunities and challenges in long-read sequencing data analysis**
Shanika L. Amarasinghe, Shian Su, Xueyi Dong, Luke Zappia, Matthew E. Ritchie, Quentin Gouil
Genome Biology (2020-02-07) https://doi.org/ggkpv7
DOI: 10.1186/s13059-020-1935-5 · PMID: 32033565 · PMCID: PMC7006217

77. **Whole-genome sequencing of eukaryotes: From sequencing of DNA fragments to a genome assembly**
K. S. Zadesenets, N. I. Ershov, N. B. Rubtsov
Russian Journal of Genetics (2017-07-12) https://doi.org/gg26n5
DOI: 10.1134/s102279541705012x

78. **Ten steps to get started in Genome Assembly and Annotation**
Victoria Dominguez Del Angel, Erik Hjerde, Lieven Sterck, Salvadors Capella-Gutierrez, Cederic Notredame, Olga Vinnere Pettersson, Joelle Amselem, Laurent Bouri, Stephanie Bocs, Christophe Klopp, … Henrik Lantz
F1000Research (2018-02-05) https://doi.org/gdq9zj
DOI: 10.12688/f1000research.13598.1 · PMID: 29568489 · PMCID: PMC5850084

79. **Whole-genome sequencing approaches for conservation biology: Advantages, limitations and practical recommendations**
Angela P. Fuentes-Pardo, Daniel E. Ruzzante
Molecular Ecology (2017-10) https://doi.org/gfzn9r
DOI: 10.1111/mec.14264 · PMID: 28746784

80. **Bioinformatic processing of RAD-seq data dramatically impacts downstream population genetic inference**
Aaron B. A. Shafer, Claire R. Peart, Sergio Tusso, Inbar Maayan, Alan Brelsford, Christopher W. Wheat, Jochen B. W. Wolf
Methods in Ecology and Evolution (2017-08) https://doi.org/gbrdv5
DOI: 10.1111/2041-210x.12700

81. **Selecting RAD-Seq Data Analysis Parameters for Population Genetics: The More the Better?**
Natalia Díaz-Arce, Naiara Rodríguez-Ezpeleta
Frontiers in Genetics (2019-05-29) https://doi.org/gg26n7
DOI: 10.3389/fgene.2019.00533 · PMID: 31191624 · PMCID: PMC6549478

82. **Harnessing the power of RADseq for ecological and evolutionary genomics**
Kimberly R. Andrews, Jeffrey M. Good, Michael R. Miller, Gordon Luikart, Paul A. Hohenlohe
Nature Reviews Genetics (2016-01-05) https://doi.org/gd85wf
DOI: 10.1038/nrg.2015.28 · PMID: 26729255 · PMCID: PMC4823021

83. **Unbroken: RADseq remains a powerful tool for understanding the genetics of adaptation in natural populations**
Julian M. Catchen, Paul A. Hohenlohe, Louis Bernatchez, W. Chris Funk, Kimberly R. Andrews, Fred W. Allendorf
Molecular Ecology Resources (2017-05) https://doi.org/f92pff
DOI: 10.1111/1755-0998.12669 · PMID: 28319339

84. **Responsible RAD: Striving for best practices in population genomic studies of adaptation**
David B. Lowry, Sean Hoban, Joanna L. Kelley, Katie E. Lotterhos, Laura K. Reed, Michael F. Antolin, Andrew Storfer

Molecular Ecology Resources (2017-05) https://doi.org/gfzn6h
DOI: 10.1111/1755-0998.12677 · PMID: 28382730

85. **Design and computational analysis of single-cell RNA-sequencing experiments**
Rhonda Bacher, Christina Kendziorski
Genome Biology (2016-04-07) https://doi.org/gc958g
DOI: 10.1186/s13059-016-0927-y · PMID: 27052890 · PMCID: PMC4823857

86. **A practical guide to single-cell RNA-sequencing for biomedical research and clinical applications**
Ashraful Haque, Jessica Engel, Sarah A. Teichmann, Tapio Lönnberg
Genome Medicine (2017-08-18) https://doi.org/gfgppz
DOI: 10.1186/s13073-017-0467-4 · PMID: 28821273 · PMCID: PMC5561556

87. **Developing a modern data workflow for evolving data**
Glenda M. Yenni, Erica M. Christensen, Ellen K. Bledsoe, Sarah R. Supp, Renata M. Diaz, Ethan P. White, S. K. Morgan Ernest
bioRxiv (2018-07-24) https://doi.org/gdqbzn
DOI: 10.1101/344804

88. **figshare - credit for all your research** https://figshare.com/

89. **Dryad Home - Publish and Preserve your Data** https://datadryad.org/stash

90. **RClone: a package to identify MultiLocus Clonal Lineages and handle clonal data sets in .**
Diane Bailleul, Solenn Stoeckel, Sophie Arnaud-Haond
Methods in Ecology and Evolution (2016-08) https://doi.org/f82t65
DOI: 10.1111/2041-210x.12550

91. **SRA in the Cloud** https://www.ncbi.nlm.nih.gov/sra/docs/sra-cloud/

92. **Cyberduck | Libre server and cloud storage browser for Mac and Windows with support for FTP, SFTP, WebDAV, Amazon S3, OpenStack Swift, Backblaze B2, Microsoft Azure & OneDrive, Google Drive and Dropbox** https://cyberduck.io/

93. **Babraham Bioinformatics - FastQC A Quality Control tool for High Throughput Sequence Data** https://www.bioinformatics.babraham.ac.uk/projects/fastqc/

94. **Integrative Genomics Viewer (IGV): high-performance genomics data visualization and exploration**
H. Thorvaldsdottir, J. T. Robinson, J. P. Mesirov
Briefings in Bioinformatics (2012-04-19) https://doi.org/f4sc43
DOI: 10.1093/bib/bbs017 · PMID: 22517427 · PMCID: PMC3603213

95. **Salmon provides fast and bias-aware quantification of transcript expression**
Rob Patro, Geet Duggal, Michael I Love, Rafael A Irizarry, Carl Kingsford
Nature Methods (2017-03-06) https://doi.org/gcw9f5
DOI: 10.1038/nmeth.4197 · PMID: 28263959 · PMCID: PMC5600148

96. **MultiQC: summarize analysis results for multiple tools and samples in a single report**
Philip Ewels, Måns Magnusson, Sverker Lundin, Max Käller
Bioinformatics (2016-10-01) https://doi.org/f3s996
DOI: 10.1093/bioinformatics/btw354 · PMID: 27312411 · PMCID: PMC5039924

97. **Identifying and mitigating bias in next-generation sequencing methods for chromatin biology**
Clifford A. Meyer, X. Shirley Liu
Nature Reviews Genetics (2014-09-16) https://doi.org/ggd9m9
DOI: 10.1038/nrg3788 · PMID: 25223782 · PMCID: PMC4473780

98. **The impact of amplification on differential expression analyses by RNA-seq**
Swati Parekh, Christoph Ziegenhain, Beate Vieth, Wolfgang Enard, Ines Hellmann
Scientific Reports (2016-05-09) https://doi.org/f8kzcp
DOI: 10.1038/srep25533 · PMID: 27156886 · PMCID: PMC4860583

99. **Detection and Removal of PCR Duplicates in Population Genomic ddRAD Studies by Addition of a Degenerate Base Region (DBR) in Sequencing Adapters**
Hannah Schweyen, Andrey Rozenberg, Florian Leese
The Biological Bulletin (2014-10) https://doi.org/f6q76c
DOI: 10.1086/bblv227n2p146 · PMID: 25411373

100. **Elimination of PCR duplicates in RNA-seq and small RNA-seq using unique molecular identifiers**
Yu Fu, Pei-Hsuan Wu, Timothy Beane, Phillip D. Zamore, Zhiping Weng
BMC Genomics (2018-07-13) https://doi.org/ggv5zp
DOI: 10.1186/s12864-018-4933-1 · PMID: 30001700 · PMCID: PMC6044086

101. **Biased estimates of clonal evolution and subclonal heterogeneity can arise from PCR duplicates in deep sequencing experiments**
Erin N Smith, Kristen Jepsen, Mahdieh Khosroheidari, Laura Z Rassenti, Matteo D'Antonio, Emanuela M Ghia, Dennis A Carson, Catriona HM Jamieson, Thomas J Kipps, Kelly A Frazer
Genome Biology (2014-08-07) https://doi.org/ggfhv7
DOI: 10.1186/s13059-014-0420-4 · PMID: 25103687 · PMCID: PMC4165357

102. **Evidence for extensive horizontal gene transfer from the draft genome of a tardigrade**
Thomas C. Boothby, Jennifer R. Tenlen, Frank W. Smith, Jeremy R. Wang, Kiera A. Patanella, Erin Osborne Nishimura, Sophia C. Tintori, Qing Li, Corbin D. Jones, Mark Yandell, … Bob Goldstein
Proceedings of the National Academy of Sciences (2015-12-29) https://doi.org/9gs
DOI: 10.1073/pnas.1510461112 · PMID: 26598659 · PMCID: PMC4702960

103. **No evidence for extensive horizontal gene transfer in the genome of the tardigrade *Hypsibius dujardini***
Georgios Koutsovoulos, Sujai Kumar, Dominik R. Laetsch, Lewis Stevens, Jennifer Daub, Claire Conlon, Habib Maroon, Fran Thomas, Aziz A. Aboobaker, Mark Blaxter
Proceedings of the National Academy of Sciences (2016-05-03) https://doi.org/f8nrtb
DOI: 10.1073/pnas.1600338113 · PMID: 27035985 · PMCID: PMC4983863

104. **Large-scale contamination of microbial isolate genomes by Illumina PhiX control**
Supratim Mukherjee, Marcel Huntemann, Natalia Ivanova, Nikos C Kyrpides, Amrita Pati
Standards in Genomic Sciences (2015-03-30) https://doi.org/ggv5zn
DOI: 10.1186/1944-3277-10-18 · PMID: 26203331 · PMCID: PMC4511556

105. **Index hopping on the Illumina HiseqX platform and its consequences for ancient DNA studies**
Tom van der Valk, Francesco Vezzi, Mattias Ormestad, Love Dalén, Katerina Guschanski
Molecular Ecology Resources (2019-05-05) https://doi.org/gfwtvw
DOI: 10.1111/1755-0998.13009 · PMID: 30848092

106. **On the optimal trimming of high-throughput mRNA sequence data**
Matthew D. MacManes
Frontiers in Genetics (2014) https://doi.org/gfn5g7
DOI: 10.3389/fgene.2014.00013 · PMID: 24567737 · PMCID: PMC3908319

107. **Streaming histogram sketching for rapid microbiome analytics**
Will PM Rowe, Anna Paola Carrieri, Cristina Alcon-Giner, Shabhonam Caim, Alex Shaw, Kathleen Sim, J. Simon Kroll, Lindsay J. Hall, Edward O. Pyzer-Knapp, Martyn D. Winn
Microbiome (2019-03-16) https://doi.org/ggv5zq
DOI: 10.1186/s40168-019-0653-2 · PMID: 30878035 · PMCID: PMC6420756

108. **When the levee breaks: a practical guide to sketching algorithms for processing the flood of genomic data**
Will P. M. Rowe
Genome Biology (2019-09-13) https://doi.org/gf8bfj
DOI: 10.1186/s13059-019-1809-x · PMID: 31519212 · PMCID: PMC6744645

109. **will-rowe/genome-sketching**
GitHub
https://github.com/will-rowe/genome-sketching

110. **STAR: ultrafast universal RNA-seq aligner**
Alexander Dobin, Carrie A. Davis, Felix Schlesinger, Jorg Drenkow, Chris Zaleski, Sonali Jha, Philippe Batut, Mark Chaisson, Thomas R. Gingeras
Bioinformatics (2013-01) https://doi.org/f4h523
DOI: 10.1093/bioinformatics/bts635 · PMID: 23104886 · PMCID: PMC3530905

111. **Graph-based genome alignment and genotyping with HISAT2 and HISAT-genotype**
Daehwan Kim, Joseph M. Paggi, Chanhee Park, Christopher Bennett, Steven L. Salzberg
Nature Biotechnology (2019-08-02) https://doi.org/gf5395
DOI: 10.1038/s41587-019-0201-4 · PMID: 31375807

112. **Near-optimal probabilistic RNA-seq quantification**
Nicolas L Bray, Harold Pimentel, Páll Melsted, Lior Pachter
Nature Biotechnology (2016-04-04) https://doi.org/f8nvsp
DOI: 10.1038/nbt.3519 · PMID: 27043002

113. **RapMap: a rapid, sensitive and accurate tool for mapping RNA-seq reads to transcriptomes**
Avi Srivastava, Hirak Sarkar, Nitish Gupta, Rob Patro
Bioinformatics (2016-06-15) https://doi.org/f8vm2j
DOI: 10.1093/bioinformatics/btw277 · PMID: 27307617 · PMCID: PMC4908361

114. **The Types, Roles, and Practices of Documentation in Data Analytics Open Source Software Libraries**
R. Stuart Geiger, Nelle Varoquaux, Charlotte Mazel-Cabasse, Chris Holdgraf
Computer Supported Cooperative Work (CSCW) (2018-05-29) https://doi.org/gd4rhr
DOI: 10.1007/s10606-018-9333-1

115. **Data Carpentry: Workshops to Increase Data Literacy for Researchers**
Tracy K. Teal, Karen A. Cranston, Hilmar Lapp, Ethan White, Greg Wilson, Karthik Ram, Aleksandra Pawlik
International Journal of Digital Curation (2015-03-18) https://doi.org/gf5hjw
DOI: 10.2218/ijdc.v10i1.351

116. **BioStar: An Online Question & Answer Resource for the Bioinformatics Community**
Laurence D. Parnell, Pierre Lindenbaum, Khader Shameer, Giovanni Marco Dall'Olio, Daniel C. Swan, Lars Juhl Jensen, Simon J. Cockell, Brent S. Pedersen, Mary E. Mangan, Christopher A. Miller, Istvan Albert
PLoS Computational Biology (2011-10-27) https://doi.org/dhsz4k
DOI: 10.1371/journal.pcbi.1002216 · PMID: 22046109 · PMCID: PMC3203049

117. **How to create a Minimal, Reproducible Example - Help Center**
Stack Overflow
https://stackoverflow.com/help/minimal-reproducible-example

118. **FAQ: How to do a minimal reproducible example (reprex) for beginners**
RStudio Community
(2020-03-25) https://community.rstudio.com/t/faq-how-to-do-a-minimal-reproducible-example-reprex-for-beginners/23061

119. **Code of conduct in open source projects**
Parastou Tourani, Bram Adams, Alexander Serebrenik
Institute of Electrical and Electronics Engineers (IEEE) (2017-02) https://doi.org/gfzbs6
DOI: 10.1109/saner.2017.7884606

120. **Building a local community of practice in scientific programming for life scientists**
Sarah L. R. Stevens, Mateusz Kuzak, Carlos Martinez, Aurelia Moser, Petra Bleeker, Marc Galland
PLOS Biology (2018-11-28) https://doi.org/gfpwkb
DOI: 10.1371/journal.pbio.2005561 · PMID: 30485260 · PMCID: PMC6287879

Click here to access/download;Figure;Figure1-workflows.pdf

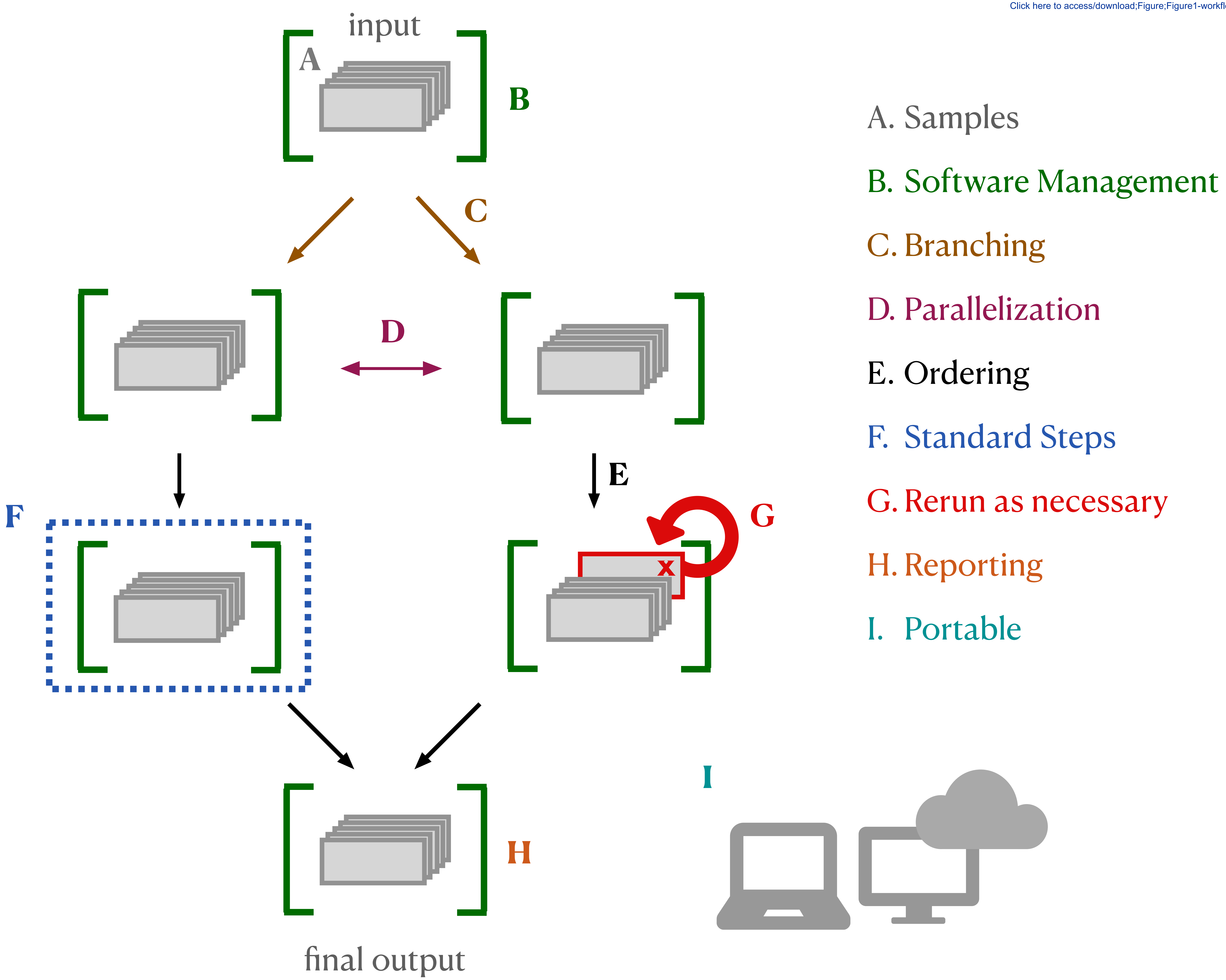

 Click here to access/download;Figure;Figure2-conda.pdf

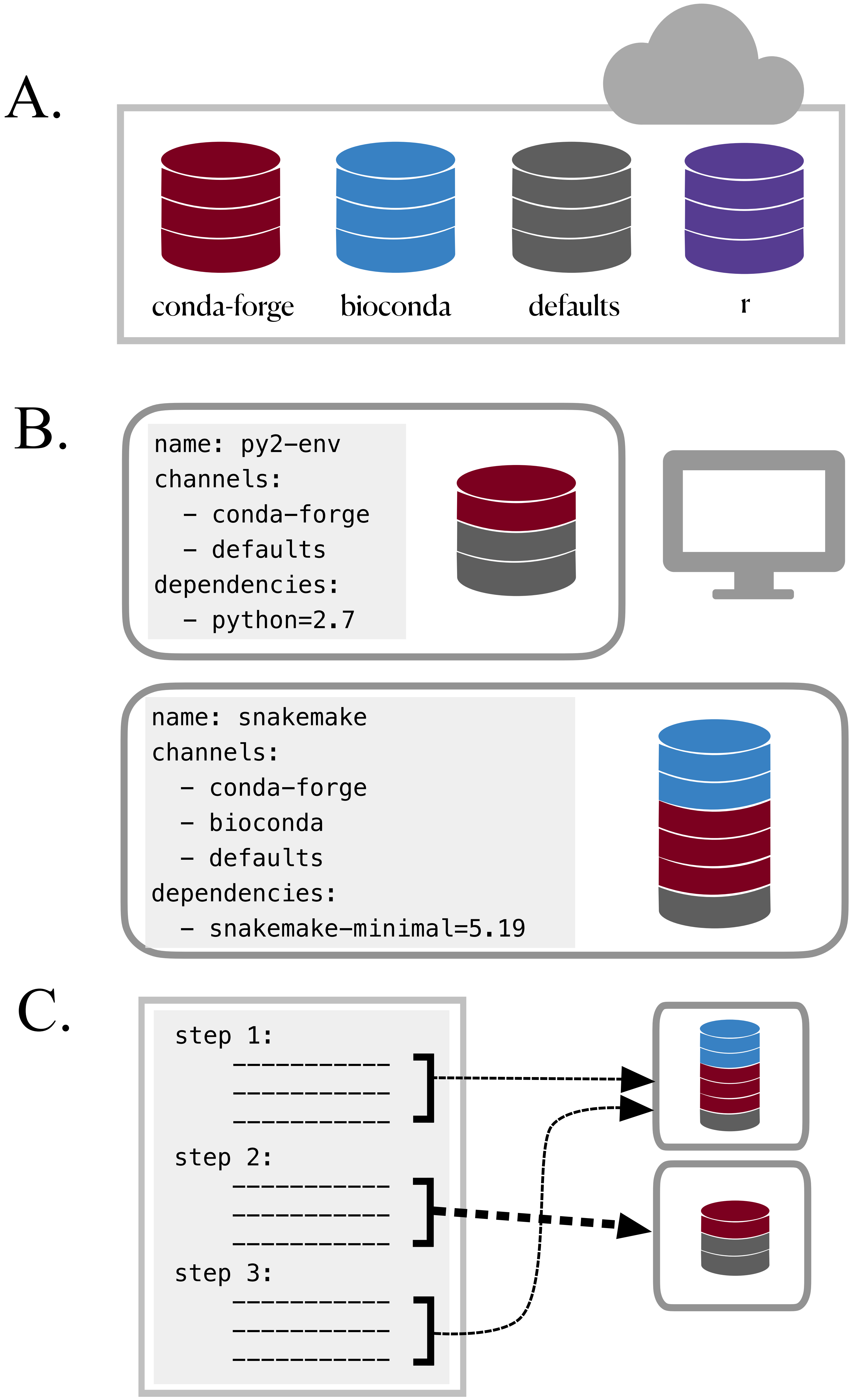

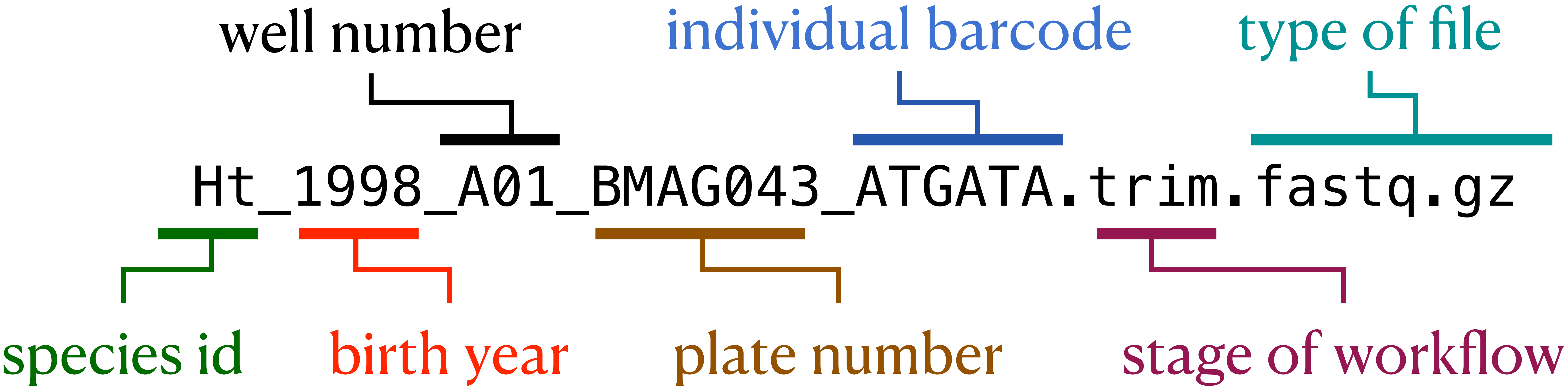
well number
individual barcode
type of file
Ht_1998_A01_BMAG043_ATGATA.trim.fastq.gz
species id
birth year
plate number
stage of workflow

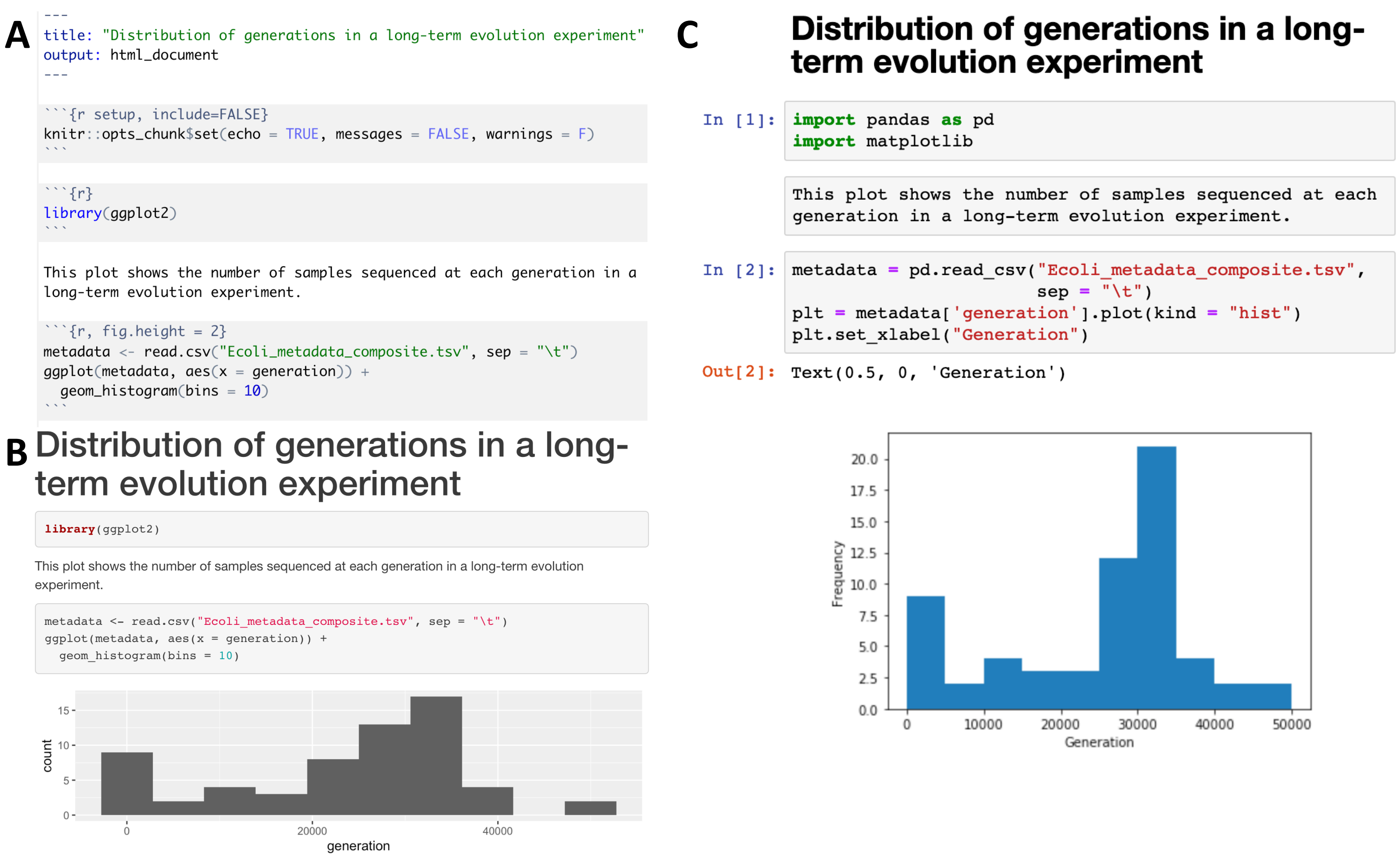
A
---
title: "Distribution of generations in a long-term evolution experiment"
output: html_document

```{r setup, include=FALSE}
knitr::opts_chunk$set(echo = TRUE, messages = FALSE, warnings = F)
```
```{r}
library(ggplot2)
```
This plot shows the number of samples sequenced at each generation in a long-term evolution experiment.
```{r, fig.height = 2}
metadata <- read.csv("Ecoli_metadata_composite.tsv", sep = "\t")
ggplot(metadata, aes(x = generation)) +
geom_histogram(bins = 10)
```
B
Distribution of generations in a long-term evolution experiment
library(ggplot2)
This plot shows the number of samples sequenced at each generation in a long-term evolution experiment.
metadata <- read.csv("Ecoli_metadata_composite.tsv", sep = "\t")
ggplot(metadata, aes(x = generation)) +
geom_histogram(bins = 10)
count
0
5
10
15
0
20000
40000
generation
C
Distribution of generations in a long-term evolution experiment
In [1]:
import pandas as pd
import matplotlib
This plot shows the number of samples sequenced at each generation in a long-term evolution experiment.
In [2]:
metadata = pd.read_csv("Ecoli_metadata_composite.tsv",
sep = "\t")
plt = metadata['generation'].plot(kind = "hist")
plt.set_xlabel("Generation")
Out[2]:
Text(0.5, 0, 'Generation')
Frequency
0.0
2.5
5.0
7.5
10.0
12.5
15.0
17.5
20.0
0
10000
20000
30000
40000
50000
Generation

Figure 5: rnaseq dag

Click here to access/download;Figure;Figure5-rnaseq-dag.pdf

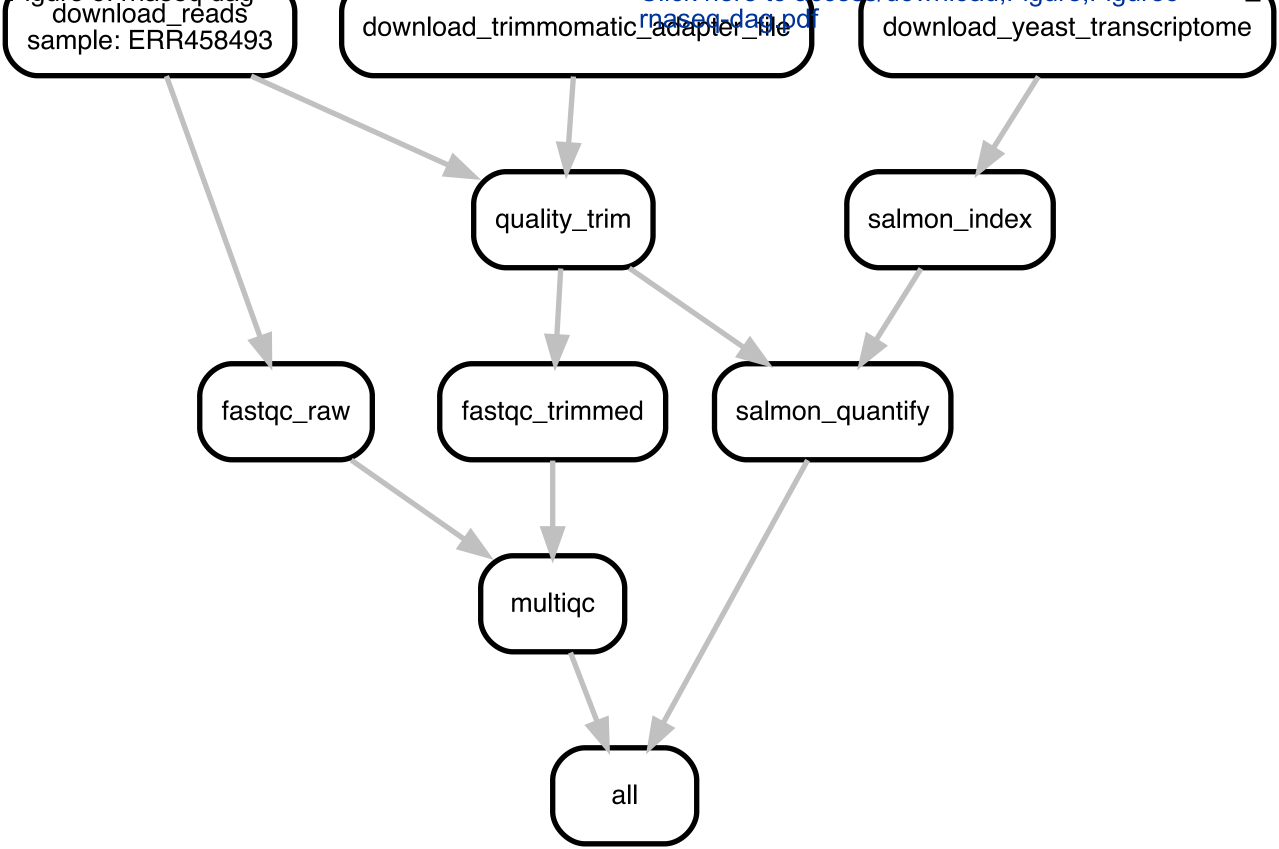

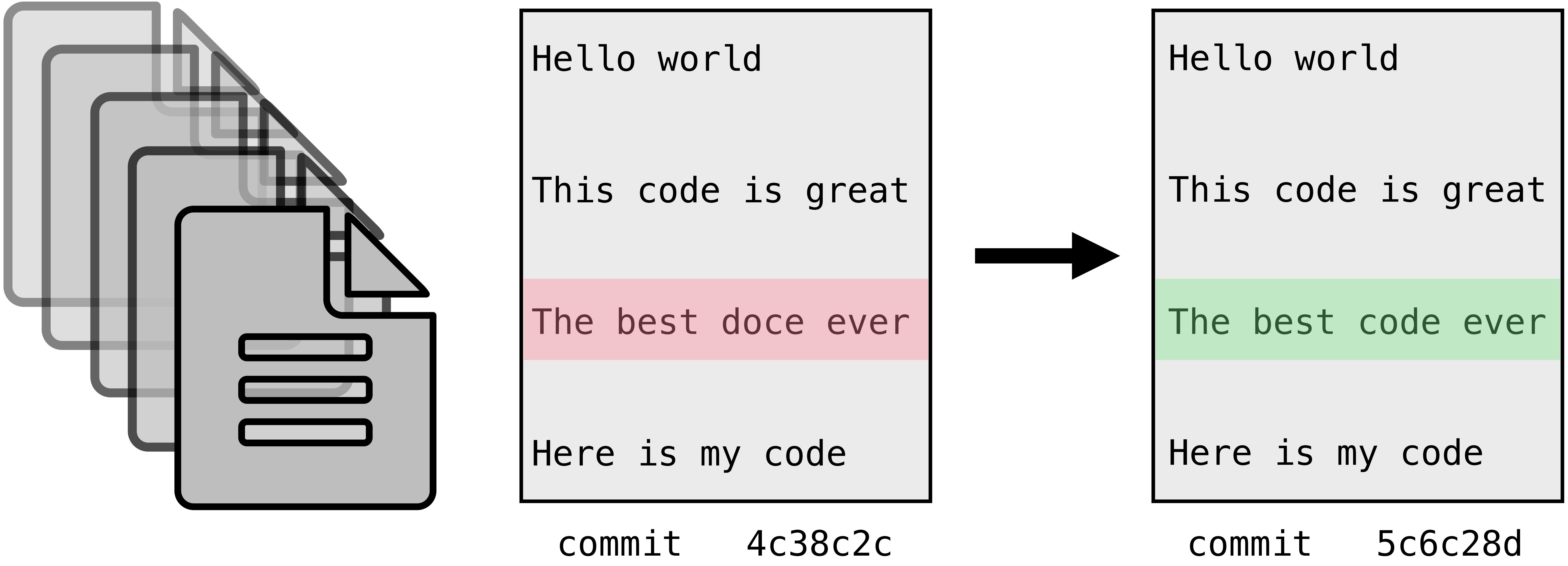
Hello world
This code is great
The best doce ever
Here is my code
commit 4c38c2c
Hello world
This code is great
The best code ever
Here is my code
commit 5c6c28d

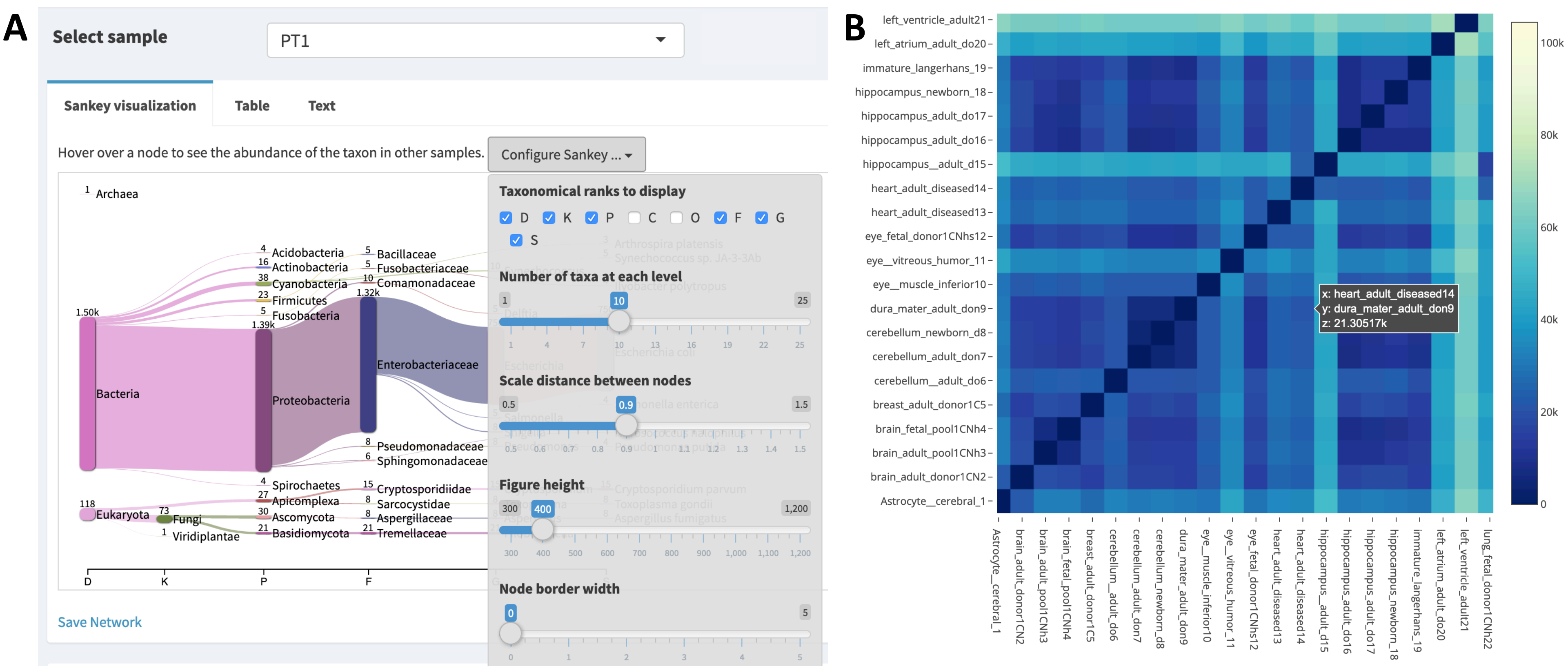
A
Select sample
PT1
Sankey visualization
Table
Text
Hover over a node to see the abundance of the taxon in other samples.
Configure Sankey ...
Taxonomical ranks to display
D K P C O F G S
Number of taxa at each level
Scale distance between nodes
Figure height
Node border width
Archaea
Bacteria
Proteobacteria
Enterobacteriaceae
Acidobacteria
Actinobacteria
Cyanobacteria
Firmicutes
Fusobacteria
Bacillaceae
Fusobacteriaceae
Comamonadaceae
Pseudomonadaceae
Sphingomonadaceae
Eukaryota
Fungi
Viridiplantae
Spirochaetes
Apicomplexa
Ascomycota
Basidiomycota
Cryptosporidiidae
Sarcocystidae
Aspergillaceae
Tremellaceae
Save Network
B
left_ventricle_adult21
left_atrium_adult_do20
immature_langerhans_19
hippocampus_newborn_18
hippocampus_adult_do17
hippocampus_adult_do16
hippocampus__adult_d15
heart_adult_diseased14
heart_adult_diseased13
eye_fetal_donor1CNhs12
eye__vitreous_humor_11
eye__muscle_inferior10
dura_mater_adult_don9
cerebellum_newborn_d8
cerebellum_adult_don7
cerebellum__adult_do6
breast_adult_donor1C5
brain_fetal_pool1CNh4
brain_adult_pool1CNh3
brain_adult_donor1CN2
Astrocyte__cerebral_1
lung_fetal_donor1CNh22
x: heart_adult_diseased14
y: dura_mater_adult_don9
z: 21.30517k
100k
80k
60k
40k
20k
0

Figure 8: checksum-file

Click here to access/download;Figure;Figure8-checksum-file.pdf

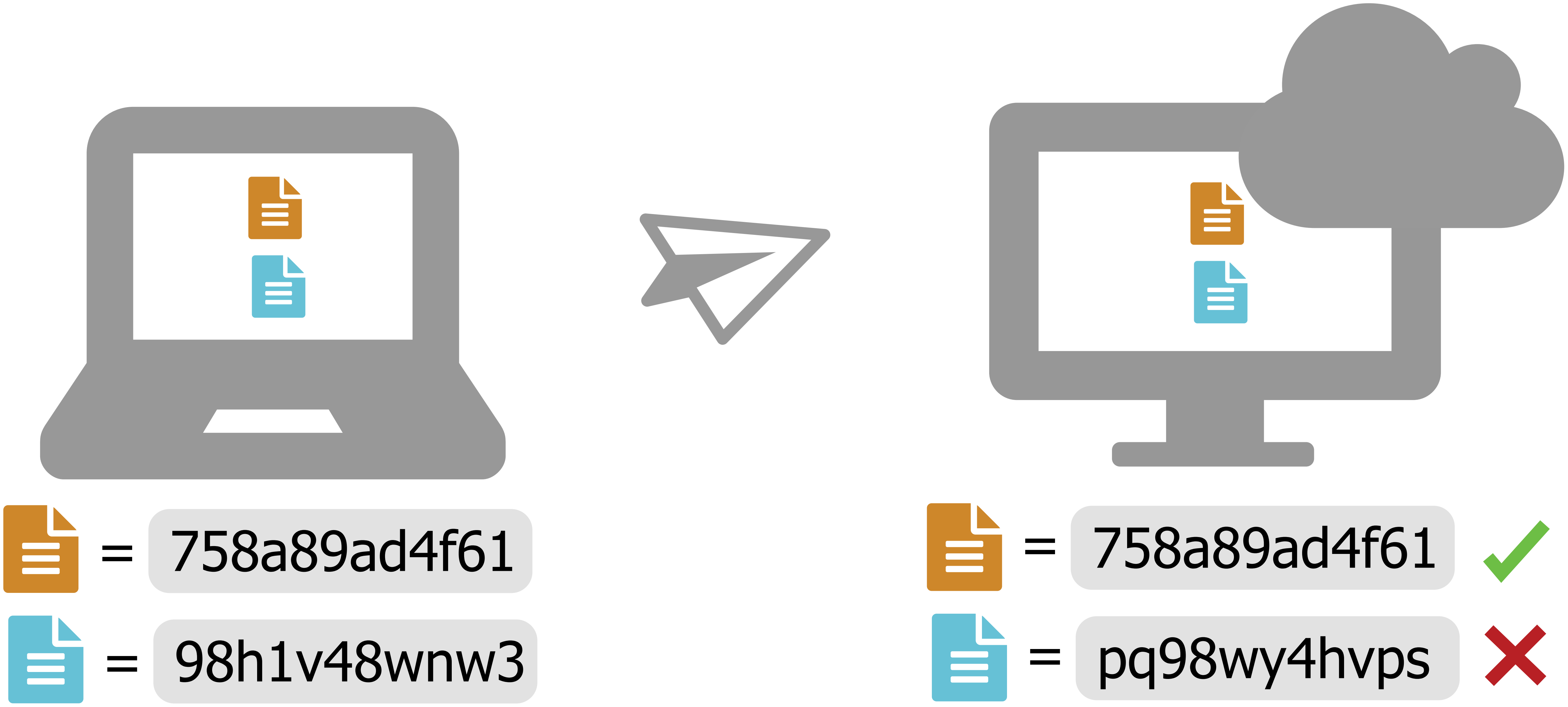

Figure 9: multiqc-viz
Click here to access/download;Figure;Figure9-multiqc-viz.pdf

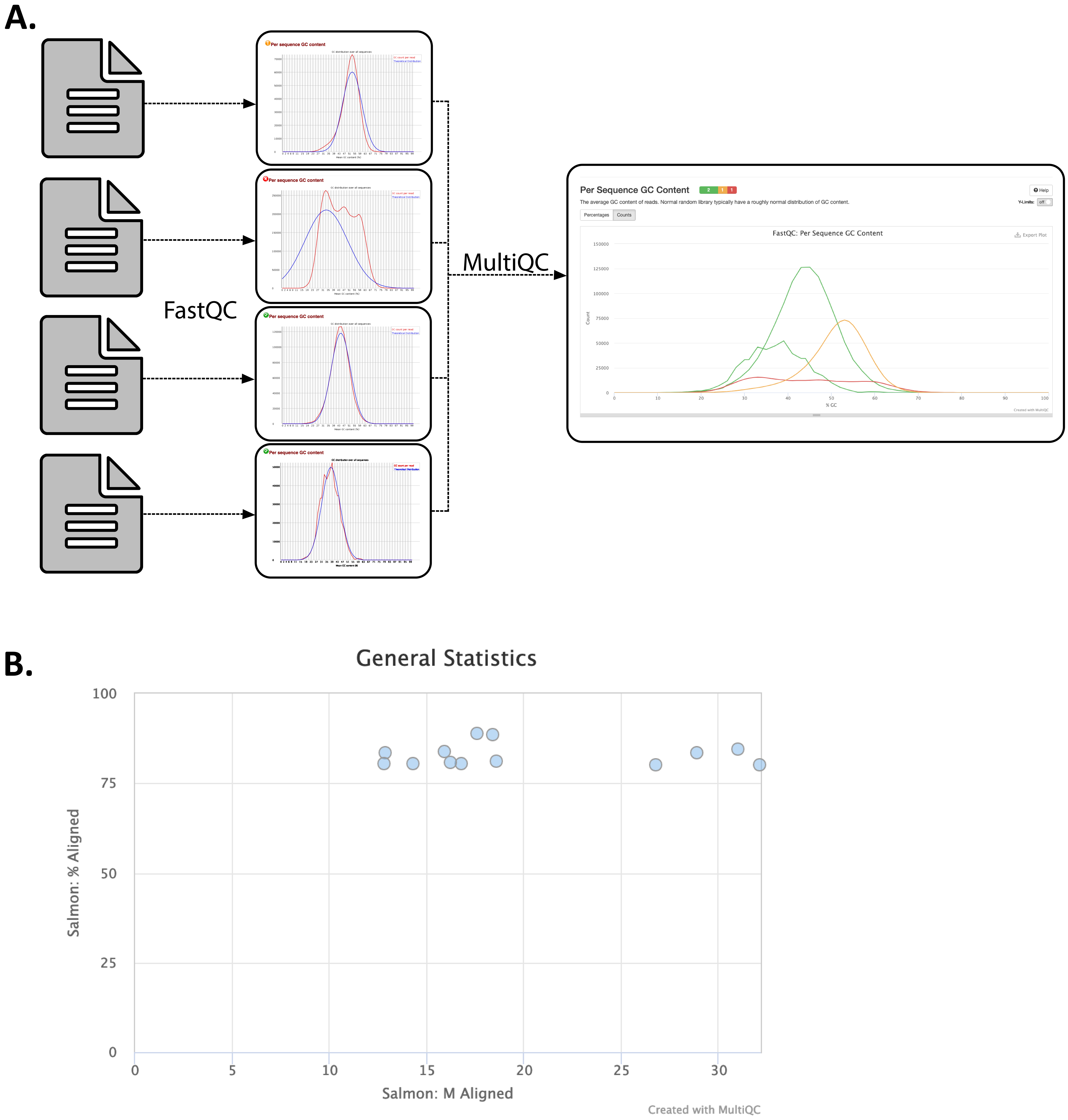

pdf version of manuscript (figures embedded)

Click here to access/download
Supplementary Material
2020-workflows-08-2020.pdf

Cover Letter
Click here to access/download;Personal Cover;2020-workflows-paper-cover-letter-8-2020-signed.pdf

UNIVERSITY OF CALIFORNIA, DAVIS

BERKELEY • DAVIS • IRVINE • LOS ANGELES • RIVERSIDE • SAN DIEGO • SAN FRANCISCO SANTA BARBARA • SANTA CRUZ

DEPARTMENT OF POPULATION HEALTH
AND REPRODUCTION
SCHOOL OF VETERINARY MEDICINE
(530) 752-1358
FAX (530) 752-4278

ONE SHIELDS AVENUE
DAVIS, CALIFORNIA 95616-8743

Dear Editors,

Enclosed please find our manuscript entitled **"Streamlining Data-Intensive Biology with Workflow Systems"** by Taylor Reiter, Phillip T. Brooks, Luiz Irber, Shannon E.K. Joslin, Charles M. Reid, Camille Scott, C. Titus Brown, and N. Tessa Pierce, for consideration for publication in GigaScience. Our pre-print is on biorxiv (https://doi.org/10.1101/2020.06.30.178673), but this article is not submitted elsewhere.

This manuscript provides a directed set of project, data, and resource management strategies for adopting computational workflow systems to facilitate and expedite reproducible research in biology.

The biological research community has expressed a strong commitment to the idea that all life sciences research - including data and analysis workflows - should be Findable, Accessible, Interoperable, and Reusable (FAIR). These ideals are readily achievable for computational analyses, but implementing them in practice has proven difficult, particularly for biologists with limited computational training. Data-centric workflow systems offer a solution by internally managing computational resources, software, and conditional execution of analysis steps using a declarative specification of the workflow. These systems are reshaping the landscape of biological data analysis and empowering researchers to conduct reproducible analyses at scale. Online communities for sharing reusable workflow code have proliferated, mitigating the initial costs of learning and implementing workflow code, enhancing reproducibility, and leading to faster time-to-insight.

There is broad interest in workflows for biological analyses, recently profiled in Nature Methods ("When Computational Pipelines go 'clank' ") and Nature Toolbox ("Workflow systems turn raw data into scientific knowledge"; see citations at end of letter), but few practical resources exist. In this manuscript, we build upon strong "best" and "good-enough" practice recommendations for biological computing (articles below) to **provide what we believe to be the first detailed guidance for employing actual workflow systems in data-intensive biology research**.

The following three articles are most similar to ours, each providing specific guidance for biological computing. None address workflow systems.

> Wilson G, Bryan J, Cranston K, Kitzes J, Nederbragt L, Teal TK (2017) Good enough practices in scientific computing. PLoS Comput Biol 13(6): e1005510. https://doi.org/10.1371/journal.pcbi.1005510

Shade A, Teal TK (2015) Computing Workflows for Biologists: A Roadmap. PLoS Biol 13(11): e1002303. https://doi.org/10.1371/journal.pbio.1002303

Wilson G, Aruliah DA, Brown CT, Chue Hong NP, Davis M, Guy RT, et al. (2014) Best Practices for Scientific Computing. PLoS Biol 12(1): e1001745. https://doi.org/10.1371/journal.pbio.1001745

Data-centric workflow systems are expanding our capacity to conduct end-to-end FAIR analyses in computational biology, maximizing the value of both analysis code and generated data. The strategies presented here are designed to accelerate structured adoption of these systems for the benefit of individual researchers and the research community as a whole.

Thank you for your consideration.

Sincerely,

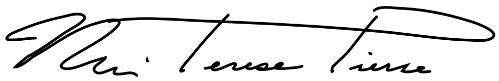

N. Tessa Pierce (corresponding author)
NSF Postdoctoral Fellow
Department of Population Health and Reproduction
University of California, Davis

Additional citations:

Marx, Vivien. "When computational pipelines go ‘clank’." Nature Methods (2020): 1-4. https://doi.org/10.1038/s41592-020-0886-9

Perkel, Jeffrey M. "Workflow systems turn raw data into scientific knowledge." Nature 573.7772 (2019): 149-150. http://dx.doi.org/10.1038/d41586-019-02619-z

Suggested Reviewers:

Neil Chue Hong
Software Sustainability Institute
n.chuehong@epcc.ed.ac.uk

Kate Hertweck, PhD
Fred Hutchinson Cancer Research Center
k8hertweck@gmail.com

Greg Wilson, PhD
RStudio
greg.wilson@rstudio.com

Karen Cranston, PhD
Agriculture and Agri-Food Canada
karen.cranston@gmail.com

Ethan White, PhD
University of Florida
ethanwhite@ufl.edu
