# Supplementary material for: Streamlining data-intensive biology with workflow systems
Source: Gigascience. 2021 Jan 13;10(1):giaa140. doi: 10.1093/gigascience/giaa140 (PMC8631065; doi:10.1093/gigascience/giaa140)
Supplement: giaa140_Response_to_Reviewer_Comments_Original_Submission [file giaa140_response_to_reviewer_comments_original_submission.pdf]

Author's Response To Reviewer Comments

Close

Dear Editors,

Thank you for the opportunity to submit a revised draft of our manuscript "Streamlining Data-Intensive Biology with Workflow Systems" by Reiter et al. We appreciate the effort you and the reviewers have dedicated to providing valuable feedback for improving the manuscript. In particular, we thank the reviewers for their strong points on managing reproducible scientific software installations. We hope that the edited manuscript better describes the important compromises we see between usability, installability, and reproducibility.

Below, please find a point-by-point response to each review. The manuscript changes are also available on github, perhaps most easily viewed here: https://github.com/dib-lab/2020-workflows-paper/pull/58/files.

Reviewer #1:
>This manuscript is a review that provides insights into creating workflows and executing them using workflow engines. The authors also provide extensive practical recommendations on performing data-intensive biology. Overall it is easy to understand, and the figures are nice. Below are some changes that I suggest.
>
>1. The Abstract states, "These workflows commonly produce hundreds to thousands of intermediate files." Can you provide an example of a workflow that would require such a large number of intermediate files? That would help to make this need more concrete.

We have better described large workflows and linked to a larger directed acyclic graph (DAG) within the Figure 5 caption.

>2. Line 76: "These features ensure that the steps for data analysis are minimally documented…" Consider rephrasing. To me it reads that minimal documentation is desirable. But I think you mean that things should be documented at least to a minimum requirement.

Yes - we have rephrased this sentence to better reflect the point.

>3. The paper briefly emphasizes software containers. It puts more emphasize on software-management systems like conda. However, in my opinion, containers are a critical tool and should be emphasized more. Software-management systems sometimes cannot install all of the required dependencies, and not all tools are included in these systems. However, containers provide more flexibility for these types of situations and are supported by all or nearly all workflow management systems.

We have reworked the software management section to better describe the range of software management solutions, including both limitations of conda and benefits and limitations of container-based software installation.

>4. Type-o on line 138: "devloping"

fixed - thanks!

>5. Line 205: "Using software without learning management systems." I'm not sure that I understand the wording on this. This term has a very specific meaning in education administration (https://en.wikipedia.org/wiki/Learning_management_system), but I think you mean something different. Also, that who section feels a bit disjointed. It was difficult to wrap my mind around exactly

what it is trying to say.

We have reworded this section for clarity. We hope it now reflects the scope of software usage and installation options and provides some useful context for how to choose between them.

>6. Line 268: Readers may be unclear what "seqanswers" is. Please provide more context.

We removed this unnecessary reference. SeqAnswers is described instead in a later portion of the manuscript.

>7. Lines 410-411: This sentence seems unnecessary. Same with lines 420-421.

Removed.

>8. Line 474: consider using italics rather than bold text to emphasize this point.

Agreed - changed.

>9. Line 502: You reference these repositories in the next paragraph, but it would be better to do so the first time you mention them.

We moved the references here, thanks.

>10. Line 509: This statement is somewhat subjective. Consider removing it or providing a more detailed justification.

We have pared down this section.

>11. Table 3: Some users may be unfamiliar with what bash is.

Removed reference to bash.

>12. Line 638: Should be "Principal Component Analysis"

fixed!

>13. The manuscript starts with a focus on using workflow systems. Later it provides a wide range of general recommendations for doing data-intensive biology. Maybe it's just me, but I felt like it got too long and a bit unfocused in the second half. It's up to you, but you might consider splitting it into two manuscripts: one on workflow systems and one on data-intensive biology. Or maybe consider removing/simplifying some of the sections so that it is not so long and is a bit more focused.

We recognize that the manuscript is long, but feel that it provides a comprehensive set of recommendations for researchers to get started using data-intensive workflows for working with sequencing data. In particular, we think that the quality control and data management recommendations we discuss in the latter half of the manuscript are critical for building high-quality, reproducible sequence analysis workflows.

Reviewer #2:
>
>Reiter et al. provides a review on the current state of the data-centric workflows for data analysis tasks in biology. The authors touch all aspects of data analysis tasks, not only the workflow systems but also the whole ecosystem that contains everything from workflow management systems to data integrity and managing computational resources. I generally found the recommendations and the review very useful for the community except one point detailed below.
>
>I understand this manuscript is based on personal experiences as authors acknowledged in their summary. However, in my opinion, this review misses important developments in reproducibility that are

compatible with the main recommendations of the review.
>
>The authors mention software wrangling as a crucial part in scientific reproducibility assuming that the initial data is intact and available. They mention Conda, Singularity, and Docker as methods to manage software for reproducibility. However, we see reproducibility as a spectrum. A fully reproducible workflow would have the following ingredients assuming the data is available: 1) code and usage transparency, 2) installability and 3) reproduction of runtime environment. The authors give reasonable recommendations for the code and usage transparency which is mainly making sure that the code is documented to the highest quality, and available publicly. Conda, Singularity and Docker remedies the installation problem. They make dependencies easily installable in most cases. However, authors do not mention short-comings of these solutions. The main short-coming of those tools is that they don't fully satisfy reproduction of the runtime environment if they do so in the case of docker/singularity they do this opaquely and are not transparent. It is hard to verify exactly what are the contents of a container. Docker recommends use of docker files but they do not necessarily have the version of the software that is in the container. It is mostly a collection of commands installing software from package managers. Which brings us to the same problems I describe for condo below. These commands often include invocations of package managers like Apt (in the case of Debian-derived foundations). A package installed via apt today will *not* be identical to the same package installed a year ago. Containers are also harder to maintain if you do not analyze data and develop code on dockerized environments exclusively.
>
>Conda packages, on the other hand, do not provide reproducibility at all. You can get different binaries for the same name+version query at different times and there is no way to track which source files of dependencies produced that binary. The system environment where the software is built is not isolated. During the build, processes have access to other libraries that are not in the package recipe and also conda assumes certain low-level packages to be available in all environments.
>
>In essence, the authors don't mention that *all* the tools they recommend (conda, docker and singularity) are completely time-dependent. Some Conda channels provide archives of pre-built binaries, yes, but since not all dependencies (beyond the kernel) are taken into account at build time you will *not* be able to run these binaries without changes on a different system.
>
>There are package managers like GNU Guix remedies most of these problems and provide the state-of-the-art reproducibility exemplified by the recent "Ten Years Reproducibility Challenge" (https://www.nature.com/articles/d41586-020-02462-7). This package management system is also incorporated in snakeMake-based pipelines providing gold-standard reproducibility for multiple NGS analysis pipelines (https://academic.oup.com/gigascience/article/7/12/giy123/5114263).
>
>Altuna Akalin & Ricardo Wurmus

We strongly agree that reproducibility is a spectrum, and thank the reviewers for their thoughtful discussion and references. In response, we've extensively revised the software management portion of the manuscript, detailing the reproducibility limitations of each approach and adding information on functional package managers like GNU guix and Nix. Additionally, we have added some information on choosing between these systems that will hopefully convey the merits of each.

In our experience, software management systems currently present significant trade-offs between usability, installability, and reproducibility. In providing recommendations to the field, we feel it is important to prioritize usability, which is a critical determinant for widespread adoption of computational techniques. Systems that provide 100% reproducibility but are not straightforward to implement are not a viable solution for researchers with limited computational training or experience.

While conda is not currently the best system for producing long-term (10-year) reproducibility, we feel that it provides a reasonable balance between usability and reproducibility, particularly when used according to the provided recommendations (small, isolated, version-pinned environments are critical for both usability and reproducibility). Furthermore, broad community adoption has resulted in proliferation of conda-installable tools and helps ensure that the ecosystem around these tools will be maintained and improved over the coming years.

The expanded text on software management systems is reproduced here:

> "On the lightweight end, the conda package manager has emerged as a leading software management solution for research workflows (Figure 2). Conda handles both cluster permission and version conflict issues with a user-based software environment system, and features a straightforward “recipe” system which simplifies the process of making new software installable (including simple management of versions and updates). These features have led to widespread adoption within the bioinformatics community: packages for new software become quickly available, and can be installed easily across platforms. However, conda does not completely isolate software installations and aims neither for bitwise reproducibility nor long-term archiving of install packages, meaning installations will not be completely reproducible over time. Heavyweight software management systems package not only the software of interest, but also the runtime environment information, with the goal of ensuring perfect reproducibility in software installation over time. Tools such as singularity and docker [3,11,37,38] wrap software environments in “containers” that capture and reproduce the runtime environment information. Container-based management is particularly useful for systems where some dependencies may not be installable by lightweight managers. However, software installation within these containers can be limited by similar reproducibility issues, including changes in dependency installations over time. “Functional package managers” such as GNU Guix and Nix strictly require all dependency and configuration details be encoded within each software package, providing the most comprehensively reproducible installations. These have begun to be integrated into some bioinformatic tools [16], but have a steeper learning curve for independent use. In addition, standard installation of these managers requires system-wide installation permissions, requiring assistance from system administrators on most high-performance computing systems."

All changes can be most easily seen here: https://github.com/dib-lab/2020-workflows-paper/pull/58/files.

Overall, we hope the revised manuscript reflects the important points the reviewers have raised while still emphasizing accessible techniques that will move researchers towards fully automated, reproducible analyses.

Sincerely,

N. Tessa Pierce, corresponding author

Close
