# Supplementary material for: Streamlining data-intensive biology with workflow systems
Source: Gigascience. 2021 Jan 13;10(1):giaa140. doi: 10.1093/gigascience/giaa140 (PMC8631065; doi:10.1093/gigascience/giaa140)
Supplement: giaa140_Reviewer_1_Report_Original_Submission — Stephen R Piccolo, Ph.D. -- 9/8/2020 Reviewed [file giaa140_reviewer_1_report_original_submission.pdf]

Reviewer Report

Title: Streamlining Data-Intensive Biology with Workflow Systems

Version: Original Submission **Date:** 9/8/2020

Reviewer name: Stephen R Piccolo, Ph.D.

Reviewer Comments to Author:

This manuscript is a review that provides insights into creating workflows and executing them using workflow engines. The authors also provide extensive practical recommendations on performing data-intensive biology. Overall it is easy to understand, and the figures are nice. Below are some changes that I suggest.

1. The Abstract states, "These workflows commonly produce hundreds to thousands of intermediate files." Can you provide an example of a workflow that would require such a large number of intermediate files? That would help to make this need more concrete.
2. Line 76: "These features ensure that the steps for data analysis are minimally documented…" Consider rephrasing. To me it reads that minimal documentation is desirable. But I think you mean that things should be documented at least to a minimum requirement.
3. The paper briefly emphasizes software containers. It puts more emphasize on software-management systems like conda. However, in my opinion, containers are a critical tool and should be emphasized more. Software-management systems sometimes cannot install all of the required dependencies, and not all tools are included in these systems. However, containers provide more flexibility for these types of situations and are supported by all or nearly all workflow management systems.
4. Type-o on line 138: "devloping"
5. Line 205: "Using software without learning management systems." I'm not sure that I understand the wording on this. This term has a very specific meaning in education administration (https://en.wikipedia.org/wiki/Learning_management_system), but I think you mean something different. Also, that who section feels a bit disjointed. It was difficult to wrap my mind around exactly what it is trying to say.
6. Line 268: Readers may be unclear what "seqanswers" is. Please provide more context.
7. Lines 410-411: This sentence seems unnecessary. Same with lines 420-421.
8. Line 474: consider using italics rather than bold text to emphasize this point.
9. Line 502: You reference these repositories in the next paragraph, but it would be better to do so the first time you mention them.
10. Line 509: This statement is somewhat subjective. Consider removing it or providing a more detailed justification.
11. Table 3: Some users may be unfamiliar with what bash is.
12. Line 638: Should be "Principal Component Analysis"
13. The manuscript starts with a focus on using workflow systems. Later it provides a wide range of general recommendations for doing data-intensive biology. Maybe it's just me, but I felt like it got too long and a bit unfocused in the second half. It's up to you, but you might consider splitting it into two

manuscripts: one on workflow systems and one on data-intensive biology. Or maybe consider removing/simplifying some of the sections so that it is not so long and is a bit more focused.

Methods

Are the methods appropriate to the aims of the study, are they well described, and are necessary controls included? Choose an item.

Conclusions

Are the conclusions adequately supported by the data shown? Choose an item.

Reporting Standards

Does the manuscript adhere to the journal's guidelines on minimum standards of reporting? Choose an item.

Choose an item.

Statistics

Are you able to assess all statistics in the manuscript, including the appropriateness of statistical tests used? Choose an item.

Quality of Written English

Please indicate the quality of language in the manuscript: Choose an item.

Declaration of Competing Interests

Please complete a declaration of competing interests, considering the following questions:

- Have you in the past five years received reimbursements, fees, funding, or salary from an organisation that may in any way gain or lose financially from the publication of this manuscript, either now or in the future?
- Do you hold any stocks or shares in an organisation that may in any way gain or lose financially from the publication of this manuscript, either now or in the future?
- Do you hold or are you currently applying for any patents relating to the content of the manuscript?
- Have you received reimbursements, fees, funding, or salary from an organization that holds or has applied for patents relating to the content of the manuscript?
- Do you have any other financial competing interests?
- Do you have any non-financial competing interests in relation to this paper?

If you can answer no to all of the above, write 'I declare that I have no competing interests' below. If your reply is yes to any, please give details below.

I declare that I have no competing interests.

I agree to the open peer review policy of the journal. I understand that my name will be included on my report to the authors and, if the manuscript is accepted for publication, my named report including any attachments I upload will be posted on the website along with the authors' responses. I agree for my report to be made available under an Open Access Creative Commons CC-BY license (http://creativecommons.org/licenses/by/4.0/). I understand that any comments which I do not wish to be included in my named report can be included as confidential comments to the editors, which will not be published.

Choose an item.

To further support our reviewers, we have joined with Publons, where you can gain additional credit to further highlight your hard work (see: https://publons.com/journal/530/gigascience). On publication of this paper, your review will be automatically added to Publons, you can then choose whether or not to claim your Publons credit. I understand this statement.

Yes Choose an item.
