# Supplementary material for: Streamlining data-intensive biology with workflow systems
Source: Gigascience. 2021 Jan 13;10(1):giaa140. doi: 10.1093/gigascience/giaa140 (PMC8631065; doi:10.1093/gigascience/giaa140)
Supplement: giaa140_Reviewer_2_Report_Original_Submission — Altuna Akalin -- 9/26/2020 Reviewed [file giaa140_reviewer_2_report_original_submission.pdf]

**Reviewer Report**

**Title: Streamlining Data-Intensive Biology with Workflow Systems**

**Version: Original Submission** **Date:** 9/26/2020

**Reviewer name: Altuna Akalin**

**Reviewer Comments to Author:**

Reiter et al. provides a review on the current state of the data-centric workflows for data analysis tasks in biology. The authors touch all aspects of data analysis tasks, not only the workflow systems but also the whole ecosystem that contains everything from workflow management systems to data integrity and managing computational resources. I generally found the recommendations and the review very useful for the community except one point detailed below.

I understand this manuscript is based on personal experiences as authors acknowledged in their summary. However, in my opinion, this review misses important developments in reproducibility that are compatible with the main recommendations of the review.

The authors mention software wrangling as a crucial part in scientific reproducibility assuming that the initial data is intact and available. They mention Conda, Singularity, and Docker as methods to manage software for reproducibility. However, we see reproducibility as a spectrum. A fully reproducible workflow would have the following ingredients assuming the data is available: 1) code and usage transparency, 2) installability and 3) reproduction of runtime environment. The authors give reasonable recommendations for the code and usage transparency which is mainly making sure that the code is documented to the highest quality, and available publicly. Conda, Singularity and Docker remedies the installation problem. They make dependencies easily installable in most cases. However, authors do not mention short-comings of these solutions. The main short-coming of those tools is that they don't fully satisfy reproduction of the runtime environment if they do so in the case of docker/singularity they do this opaquely and are not transparent. It is hard to verify exactly what are the contents of a container. Docker recommends use of docker files but they do not necessarily have the version of the software that is in the container. It is mostly a collection of commands installing software from package managers. Which brings us to the same problems I describe for condo below. These commands often include invocations of package managers like Apt (in the case of Debian-derived foundations). A package installed via apt today will *not* be identical to the same package installed a year ago. Containers are also harder to maintain if you do not analyze data and develop code on dockerized environments exclusively.

Conda packages, on the other hand, do not provide reproducibility at all. You can get different binaries for the same name+version query at different times and there is no way to track which source files of dependencies produced that binary. The system environment where the software is built is not isolated. During the build, processes have access to other libraries that are not in the package recipe and also conda assumes certain low-level packages to be available in all environments.

In essence, the authors don't mention that *all* the tools they recommend (condo, docker and singularity) are completely

time-dependent.  Some Conda channels provide archives of pre-built
binaries, yes, but since not all dependencies (beyond the kernel) are
taken into account at build time you will *not* be able to run these
binaries without changes on a different system.
There are package managers like GNU Guix remedies most of these problems and provide the state-of-the-art reproducibility exemplified by the recent "Ten Years Reproducibility Challenge" (https://www.nature.com/articles/d41586-020-02462-7). This package management system is also incorporated in snakeMake-based pipelines providing gold-standard reproducibility for multiple NGS analysis pipelines (https://academic.oup.com/gigascience/article/7/12/giy123/5114263).
Altuna Akalin & Ricardo Wurmus

**Methods**

Are the methods appropriate to the aims of the study, are they well described, and are necessary controls included? Choose an item.

**Conclusions**

Are the conclusions adequately supported by the data shown? Choose an item.

**Reporting Standards**

Does the manuscript adhere to the journal's guidelines on minimum standards of reporting? Choose an item.

Choose an item.

**Statistics**

Are you able to assess all statistics in the manuscript, including the appropriateness of statistical tests used? Choose an item.

**Quality of Written English**

Please indicate the quality of language in the manuscript: Choose an item.

**Declaration of Competing Interests**

Please complete a declaration of competing interests, considering the following questions:

- Have you in the past five years received reimbursements, fees, funding, or salary from an organisation that may in any way gain or lose financially from the publication of this manuscript, either now or in the future?

- Do you hold any stocks or shares in an organisation that may in any way gain or lose financially from the publication of this manuscript, either now or in the future?
- Do you hold or are you currently applying for any patents relating to the content of the manuscript?
- Have you received reimbursements, fees, funding, or salary from an organization that holds or has applied for patents relating to the content of the manuscript?
- Do you have any other financial competing interests?
- Do you have any non-financial competing interests in relation to this paper?

If you can answer no to all of the above, write 'I declare that I have no competing interests' below. If your reply is yes to any, please give details below.

I declare that I have no competing interests

I agree to the open peer review policy of the journal. I understand that my name will be included on my report to the authors and, if the manuscript is accepted for publication, my named report including any attachments I upload will be posted on the website along with the authors' responses. I agree for my report to be made available under an Open Access Creative Commons CC-BY license (http://creativecommons.org/licenses/by/4.0/). I understand that any comments which I do not wish to be included in my named report can be included as confidential comments to the editors, which will not be published.

Choose an item.

To further support our reviewers, we have joined with Publons, where you can gain additional credit to further highlight your hard work (see: https://publons.com/journal/530/gigascience). On publication of this paper, your review will be automatically added to Publons, you can then choose whether or not to claim your Publons credit. I understand this statement.

Yes Choose an item.
